# The nature and organization of satellite DNAs in *Petunia hybrida*, related, and ancestral genomes

**DOI:** 10.3389/fpls.2023.1232588

**Published:** 2023-10-06

**Authors:** Osamah Alisawi, Katja R. Richert-Pöggeler, J.S. (Pat) Heslop-Harrison, Trude Schwarzacher

**Affiliations:** ^1^ Department of Plant Protection, Faculty of Agriculture, University of Kufa, Najaf, Iraq; ^2^ Department of Genetics and Genome Biology, Institute for Environmental Futures, University of Leicester, Leicester, United Kingdom; ^3^ Julius Kühn-Institut, Federal Research Centre for Cultivated Plants, Institute for Epidemiology and Pathogen Diagnostics, Braunschweig, Germany; ^4^ Key Laboratory of Plant Resources Conservation and Sustainable Utilization/Guangdong Provincial Key Laboratory of Applied Botany, South China Botanical Garden, Chinese Academy of Sciences, Guangzhou, China; ^5^ South China National Botanical Garden, Chinese Academy of Sciences, Guangzhou, China

**Keywords:** repetitive DNA sequences, tandemly repeated DNA, *Petunia*, RepeatExplorer, fluorescent *in situ* hybridization, survey genomic sequences, telomere associated sequences (TAS), satellitome

## Abstract

**Introduction:**

The garden petunia, *Petunia hybrida* (Solanaceae) is a fertile, diploid, annual hybrid species (2n=14) originating from *P. axillaris* and *P. inflata* 200 years ago. To understand the recent evolution of the *P. hybrida* genome, we examined tandemly repeated or satellite sequences using bioinformatic and molecular cytogenetic analysis.

**Methods:**

Raw reads from available genomic assemblies and survey sequences of *P. axillaris* N (*PaxiN*), *P. inflata* S6, (*PinfS6*), *P. hybrida* (*PhybR27*) and the here sequenced *P. parodii* S7 (*PparS7*) were used for graph and k-mer based cluster analysis of TAREAN and RepeatExplorer. Analysis of repeat specific monomer lengths and sequence heterogeneity of the major tandem repeat families with more than 0.01% genome proportion were complemented by fluorescent *in situ* hybridization (FISH) using consensus sequences as probes to chromosomes of all four species.

**Results:**

Seven repeat families, PSAT1, PSAT3, PSAT4, PSAT5 PSAT6, PSAT7 and PSAT8, shared high consensus sequence similarity and organisation between the four genomes. Additionally, many degenerate copies were present. FISH in *P. hybrida* and in the three wild petunias confirmed the bioinformatics data and gave corresponding signals on all or some chromosomes. PSAT1 is located at the ends of all chromosomes except the 45S rDNA bearing short arms of chromosomes II and III, and we classify it as a telomere associated sequence (TAS). It is the most abundant satellite repeat with over 300,000 copies, 0.2% of the genomes. PSAT3 and the variant PSAT7 are located adjacent to the centromere or mid-arm of one to three chromosome pairs. PSAT5 has a strong signal at the end of the short arm of chromosome III in *P. axillaris* and *P.inflata*, while in *P. hybrida* additional interstitial sites were present. PSAT6 is located at the centromeres of chromosomes II and III. PSAT4 and PSAT8 were found with only short arrays.

**Discussion:**

These results demonstrate that (i) repeat families occupy distinct niches within chromosomes, (ii) they differ in the copy number, cluster organization and homogenization events, and that (iii) the recent genome hybridization in breeding *P. hybrida* preserved the chromosomal position of repeats but affected the copy number of repetitive DNA.

## 1 Introduction

The genus *Petunia* (family Solanaceae) contains some 20 species including *Petunia axillaris* subsp. *axillaris* (syn *P. axillaris*) and *Petunia integrifolia* subsp. *inflata* (syn. *Petunia inflata*), the parental taxa of the globally cultivated horticultural fertile, diploid, annual hybrid (notho-) species *Petunia hybrida* (2n = 2x = 14). *P. hybrida* was first reported from South America in the 1830s and, during breeding, has crossed and backcrossed to *P. axillaris* and other *Petunia* species, making it more like an introgression line than a true hybrid. Although *P. inflata* contributes important genes for flower color and pollination, the number of genes originating from *P. inflata* is relatively low compared to the number from *P. axillaris*, although varying between *P. hybrida* accessions (Bombarely et al., 2016).

The genome sizes of diploid *Petunia* species range in 1C DNA content from 1.3 Gb to 1.57 Gb (Mishiba et al., 2000), compared to other species in the Solanaceae, are larger than those of potato and tomato (850–900 Mb, Tomato Genome Consortium, 2012), but smaller than those of hot pepper (Kim et al., 2014). The genome assemblies of the *P. axillaris N* and *P. inflata S6* (Bombarely et al., 2016) showed that *Petunia* genomes share the Solanaceae alpha whole genome triplication approximately 49 million years ago (Mya) before the *Petunia* branch split 30 Mya. With the base chromosome number of x = 7, the genus *Petunia* is considered a sister to the Solanaceae crown clade that includes the x = 12 genera *Solanum*, *Nicotiana*, and *Capsicum* (Särkinen et al., 2013; Bombarely et al., 2016). *P. hybrida* and its parental taxa have been a model species for molecular studies of secondary metabolite pathways, reproduction, and transposon activity (Bombarely et al., 2016); with its special position in the Solanaceae family, petunia is an interesting model to study genome and chromosome organization, and repetitive DNA composition and evolution. The dominance of the *P. axillaris* within the *P. hybrida* genome (see above) prompted us to include the other *P. axillaris* subspecies, namely, *Petunia parodii*, which is known to occupy a distinct distribution from the subspecies *axillaris* (Gerats and Strommer, 2009) in our repeateome analysis.

Plant genomes are rich in repetitive DNA sequences that can make up to 90% of genomes (see Heslop-Harrison and Schwarzacher, 2011), and most of the repeats are dispersed long terminal repeat (LTR) retroelements, including Gypsy and Copia superfamilies. Tandemly repeated or satellite (satDNA) sequences are another important class of repetitive sequences, abundant in many species, but generally at a lower genome proportion due to their smaller sequence length compared to LTR retroelements (Biscotti et al., 2015). Tandemly repeated DNA consists of monomers, often with high AT/GC nucleotide ratio, and variable lengths between a few bp to more than 1 kb within arrays consisting of tens to millions of monomers. Monomers often display preferential lengths of approximately 175 bp and 360 bp, reflecting the DNA length wrapped around nucleosomes (Henikoff et al., 2001; Heslop-Harrison and Schwarzacher, 2013). Satellite repeats forming large arrays are found in distinct locations along chromosomes and are often visible as heterochromatin, e.g., in rye and wheat (Contento et al., 2005), intercalary but far more prominent in telomeric and subtelomeric positions and have been described as telomere-associated sequences (TASs). Many peri-centromeric regions harbor tandem repeats (Melters et al., 2013), where they may include functional domains for centromere-specific CENH3 binding sites (see Gong et al., 2012; Maheshwari et al., 2017; Kirov et al., 2018). Centromere-specific satellites have been found to be species- or genus-specific but can include chromosome-specific variants (e.g., Heslop-Harrison et al., 1999; Heslop-Harrison et al., 2003; Jiang et al., 2003; Song et al., 2021; Wang et al., 2022).

With the introduction of large-scale DNA sequencing, the nature, evolutionary mechanisms, and functions of repeats can be studied across whole genomes (Novák et al., 2010; Wicker et al., 2017; Liu et al., 2018; Vondrak et al., 2020), while tandemly repeated array assembly has proved impossible with short-read technologies because of the collapse of the sequence motifs within large contigs or even BAC assemblies. Although long-molecule sequence reads are becoming beneficial (Belser et al., 2018), there are still challenges in measuring copy numbers and determining genomic locations of major arrays. Large volumes of high-quality random sequence reads of 100 bp to 350 bp are suitable for the identification of abundant tandemly repeated sequence motifs from genomes with appropriate analysis tools. Graph-based clustering of raw reads, particularly using the RepeatExplorer software tools (Novák et al., 2013), allows repeat sequence and retroelement protein motif identification. Additionally, the tandem repeat analyzer (TAREAN) is a computational pipeline that can be applied to explore tandem repeats combining clustering algorithms with k-mer analysis and assemblies (Novák et al., 2017). These bioinformatics tools have now been applied to many genomes, including Solanaceae species (e.g., *Solanum* and *Nicotiana*; Dodsworth et al., 2017; Gaiero et al., 2019; Zhou et al., 2019; de Souza et al., 2022), complementing the earlier studies on repeats (Lapitan et al., 1989; Tek et al., 2005; Lim et al., 2006; Chang et al., 2008).

In *Petunia*, DNA transposable elements have been studied, but little is known about other repeats (Shepherd et al., 1990; Richert-Pöggeler and Schwarzacher, 2009). In the *P. axillaris* and *P. inflata* assemblies (Bombarely et al., 2016), approximately 60% of the genome was repeats, with a relatively high proportion of DNA transposons and unassigned low copy sequences. Preliminary repeat searches (Schwarzacher et al., 2016, **Supplementary Note 2** to Bombarely et al., 2016) revealed the existence of short, less than 60 bp or much longer repeat units of 500–1,000 bp. A strong *in situ* hybridization signal was described at the centromeres with a retroelement-derived probe, and an interesting tandem repeat structure was found in one of the scaffolds. In order to study the composition of the repetitive DNA, particularly the tandemly organized satellite DNA sequences in detail and to identify specific motifs, here, we used published raw reads of *P. axillaris* N and *P. inflata* S6 and *P. hybrida* R27 (Bombarely et al., 2016) and new sequences for *P. parodii*, with repeat finding algorithms to study the tandemly organized repeateome or satellitome of wild and hybrid petunias. Identified repeats were used for fluorescence *in situ* hybridization (FISH) to find the chromosomal locations. Sequence abundance, location, and divergence were compared within and between genomes to understand evolutionary processes and potential consequences.

## 2 Materials and methods

### 2.1 Plant material and DNA extraction

The seeds of three wild South American *Petunia* accessions were kindly provided by Cris Kuhlemeier, University of Bern, Switzerland:

- *P. axillaris* (Lam.) Britton, Sterns & Poggenb. subsp. *axillaris* cultivar N, referred to here as *P. axillaris* N, abbreviated Paxi.
- *P. axillaris* subsp. *parodii* (Steere) Cabrera cultivar S7, referred to here as *P. parodii S7*, abbreviated Ppar.
- *P. integrifolia* (Hook.) Schinz & Thell. subsp. *inflata* cultivar S6, referred to here as *P. inflata* S6, abbreviated Pinf.

Seeds of the nothospecies *Petunia × hybrida* (Hook.) Regel (syn. *Petunia* × *atkinsiana* (Sweet) D.Don ex W.H.Baxter), referred to here as *P. hybrida*, abbreviated as Phyb, were obtained as follows:

- cultivar Rdc (also known as Rose du Ciel, or Himmelsröschen): NL Chrestensen, Erfurt, Germany;
- cultivar W138, Ronald Koes, University of Amsterdam; and
- cultivar V26, Glyn Harper, John Innes Institute, Norwich.

Seeds were germinated on soil, and plants were grown in small pots in the greenhouse. Genomic DNA was prepared from young leaves using the cetyltrimethylammonium bromide (CTAB) method (Doyle and Doyle, 1990) with some modifications.

### 2.2 DNA sequences

Genomic DNA of *P. parodii* S7 was sequenced commercially by Novogene, Hong Kong, using Illumina HiSeq-PE150 reads (16 Gb), and deposited in the Sequence Read Archive (SRA) database under accession SRR22797038. Illumina HiSeq PE125 raw reads of *P. hybrida* R27 (*PhybR27*), *P. inflata* (*PinfS6*), and *P. axillaris* (*PaxiN*) were obtained from Aureliano Bombarely, Department of Horticulture, Virginia Tech, USA. The draft genome sequences (Bombarely et al., 2016) of *P. inflata* v1.0.1 (*Peinf101*) and *P. axillaris* v1.6.2 (*Peaxi162*) were downloaded from Sol Genomics Network at https://solgenomics.net/organism/Petunia_inflata/genome and https://solgenomics.net/organism/Petunia_axillaris/genome. The Genome assembly *Peax403* (GCA_026929995.1, submitted by Kuhlemeier and Cannarozzi, 2022) was downloaded from National Center for Biotechnology Information (NCBI) in March 2023.

### 2.3 Tandem repeat analysis

The programs RepeatExplorer (RE; Novák et al., 2013) and Tandem Repeat Analyser (TAREAN) (Novák et al., 2017) were used for graph-based cluster combined with k-mer analysis to identify repetitive sequences in the raw reads (using 2 Gb for each analysis) and default parameters. The TAREAN reports showed lists of resulting clusters with a genome proportion higher than 0.01% that were classified according to repeat type. Here, we concentrated on the putative satellites with high or low confidence. The reports provide consensus monomers and their length, sequence LOGOS based on k-mer analysis, cluster graphs, and the sequence contigs that form them. These were used to design primers and FISH probes (**Supplementary Table S1**).

Repeat Explorer and TAREAN were first run with *P. hybrida* R27 raw reads, and seven satellite clusters were identified: CL43 (PSAT1), CL58 (PSAT3), CL95 (PSAT4), CL102 (PSAT5), CL114 (PSAT6), CL295 (PSAT7), and CL331 (PSAT8). The analysis was subsequently repeated together with the raw reads of *PaxiN*, *PinfS6*, and *PparS7* (**Table S2**; **Supplementary Figure S1**; **Supplementary Data 1**, **2**). For each cluster, the best, longest, representative contig was extracted for further analysis. In cases where the TAREAN report did not include an expected cluster as a putative satellite, all contigs of the RepeatExplorer output were searched with the original consensus sequence to identify the cluster and contig for extraction. A consensus sequence for each repeat in each species was derived and named to include the species designation (e.g., PhybSAT1, PaxiSAT1, PinfSSA1, and PparSAT1); these were aligned to compile the overall consensus sequences for the repeats (**Supplementary Data 3**). Each consensus sequence was checked against the raw reads to calculate mapped reads (map to reference) using 0%, 5%, 10%, and 20% mismatches (**Supplementary Table S3**). Copy numbers were calculated using 1.4 Gb as the haploid genome size. Geneious software was used for pairwise and multiple alignments and map reference. PSAT sequences from the individual species were named by adding a species identifier, e.g., PaxiSAT1, PinfSAT1, PhybSAT1, and PparSAT1 (see **Supplementary Tables S4**-**S7**).

Assemblies were searched with repeat sequences of interest to find locations within scaffolds of *Peaxi162* and *Pinf101* using BLASTN (see Schwarzacher et al., 2016) and chromosomes of *Peax403* (**Supplementary Table S8**). Sequence dot plots were generated in Geneious, Flexidot (Seibt et al., 2018)) or Dotlet (SIB Vitsl-IT 2016-2023, v0.2.0 https://dotlet.vital-it.ch/). Additionally, Repbase (Jurka et al., 2005; Bao et al., 2015), Tandem repeats finder (Benson, 1999), BLAST (Basic Local Alignment Search Tool (Altschul et al., 1990), and RepeatMasker (Smit et al., 2015: http://www.repeatmasker.org) were used for checking, finding, and characterizing repeat sequences.

Sequence LOGOS were used from TAREAN or generated from mapping reads to consensus references; the sequence logo for each tandem repeat sequence was calculated in the contigs file after the whole reads were aligned to each cluster sequence, and graph heights and coverage were 30 pixels.

### 2.4 Chromosome preparation and *in situ* hybridization

Root tips were collected from young plants and then treated with 0.2 M of 8-hydroxyquinoline for 4 hr before fixation with freshly made 96% ethanol:glacial acetic acid (3:1). Chromosome preparations were made on slides in 45% acetic acid following enzymatic digestion with pectinase and cellulase. The method of Schwarzacher and Heslop-Harrison (2000) was applied for fluorescence *in situ* hybridization and the hybridization conditions, 30%–40% formamide, and 2xSSC (300 mM of NaCl and 30 mM of sodium citrate, pH 7) at 37°C, which allows sequences with 20%–25% mismatches to form hybrids. PSAT1 was amplified with specific primers (**Table S1**) from the genomic DNA of *P. axillaris* and cloned into the pGEM-T Easy Vector (Promega, Madison, WI, USA). The 155-bp insert sequence was confirmed by Sanger sequencing (GATC Biotech Company). Insert of Clone PaxiSAT1 was labeled with biotin-11-dUTP using BioPrime Labelling System (Invitrogen, Carlsbad, CA, USA). Oligo FISH probes for the six remaining repeats (**Table S1**) were synthesized as oligonucleotides 5′-labeled directly with biotin-11-dUTP (Sigma-Aldrich, Darmstadt, Germany). The 5S rDNA probe was amplified from clone pTa794 (*Triticum aestivum*, 410 bp; Gerlach and Dyer, 1980) and labeled with digoxigenin-11-dUTP using Array CGH Genomic Labelling Module (Invitrogen).

Hybridization sites were detected by streptavidin conjugated to Alexa 594 (red fluorescence) and anti-digoxigenin conjugated with fluorescein isothiocyanate (FITC) (green fluorescence). DAPI (4′,6-diamidino-2-phenylindole) in antifade solution was used for counterstaining and mounting chromosomes. Slides were examined using a Nikon Eclipse 80i microscope, and images were captured with a DS-QiMc monochrome camera and NIS-Elements v.2.34 (Nikon, Tokyo, Japan). Adobe Photoshop CS2015.5 was used for preparing and overlaying images and hybridization signals, apart from cropping, using only functions affecting the whole image equally. For each FISH probe and species, at least 10 metaphases from two different experiments were analyzed. Chromosomes were identified by morphology and designated using Roman numerals following Schwarzacher et al. (2016), with the largest being Chr I and the unequaled armed chromosomes with 45SrDNA sites being Chrs II and III. The smallest chromosome is Chr VII, and 5S rDNA sites are located on Chr II and in some accessions also on Chr IV (designated Chr 7 by Benabdelmouna and Abirached-Darmency, 1997). For the *Peax403* assembly, Arabic numerals were used and maintained here.

### 2.5 List of sequences submitted to GenBank and datasets analyzed

PaxiSAT1 cloned sequence and the consensus sequences of the satellite repeats were deposited in GenBank under accession numbers OQ676819 for PaxiSAT1 clone (155 bp), OQ579142 for PSAT1, OQ579144 for PSAT3, OQ579145 for PSAT4, OQ579146 for PSAT5, OQ579147 for PSAT6, OQ579148 for PSAT7, and OQ579149 for PSAT8. The datasets, Supplementary data 1, 2 and 3 generated and analyzed for this study can be found as file ‘Petunia_tandemly_repeated_DNA_AlisawiRichertPoggelerHeslopHarrisonSchwarzacherAdditionalInformation_DatasetForThisStudy’ in the Private link https://figshare.com/s/1ce1559d6e7474984a14.

## 3 Results

### 3.1 Identification of tandem (satellite) repeats in *Petunia* genomes

**Figure 1:**
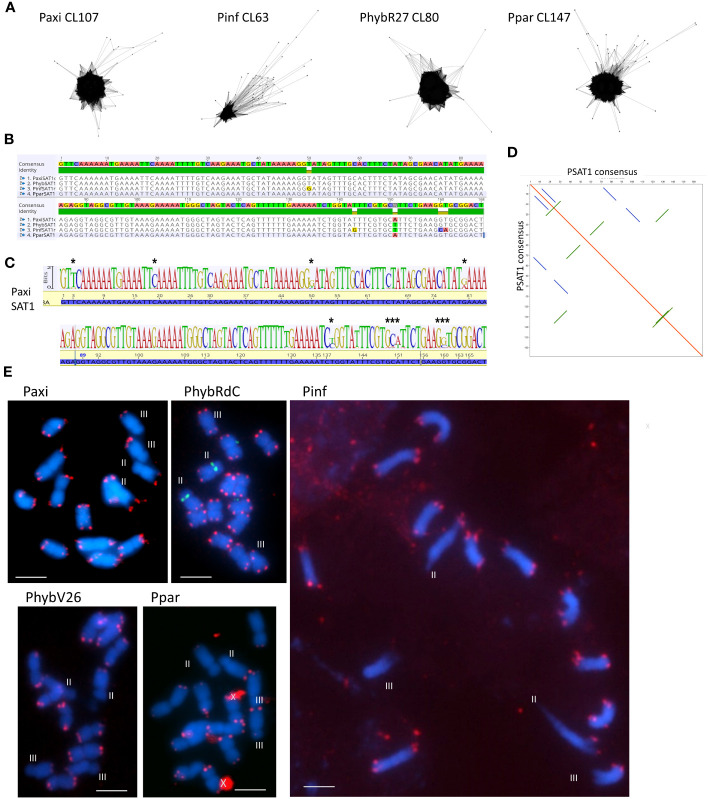
Satellite sequence PSAT1 with a 168-bp monomer. **(A)** TAREAN cluster graphs show condensed star circles. **(B)** Alignment of the species consensus sequence, PaxiSAT1, PhybSAT1, PinfSAT1, and PparSAT1 and the resulting overall consensus sequence at the top. Five nucleotides show variation between the species. **(C)** Sequence logo for PaxiSAT1 calculated by mapping the short reads to the sequence. Nucleotides with divergence are marked by asterisks (*). Compare this logo with the TAREAN logo generated from k-mer analysis (**Figure S2**). **(D)** Self dot blot of the consensus sequence (window size 12, threshold 50) showing internal direct and indirect repeats. **(E)** Fluorescence *in situ* hybridization to metaphase chromosomes (blue with DAPI) of *Petunia axillaris*, *Petunia inflata*, *Petunia hybrida* (RdC and V26), and *Petunia parodii* using the clone PSAT1 as probe (red). Hybridizations sites are visible as double dots at the end of all chromosomes but are missing on the short arms of Chrs II and III (except in PhybRdC). The 5S rDNA sites on Chr II (middle) are detected in green. Unspecific background is marked by a cross. Bar = 10 µm.

**Figure 2:**
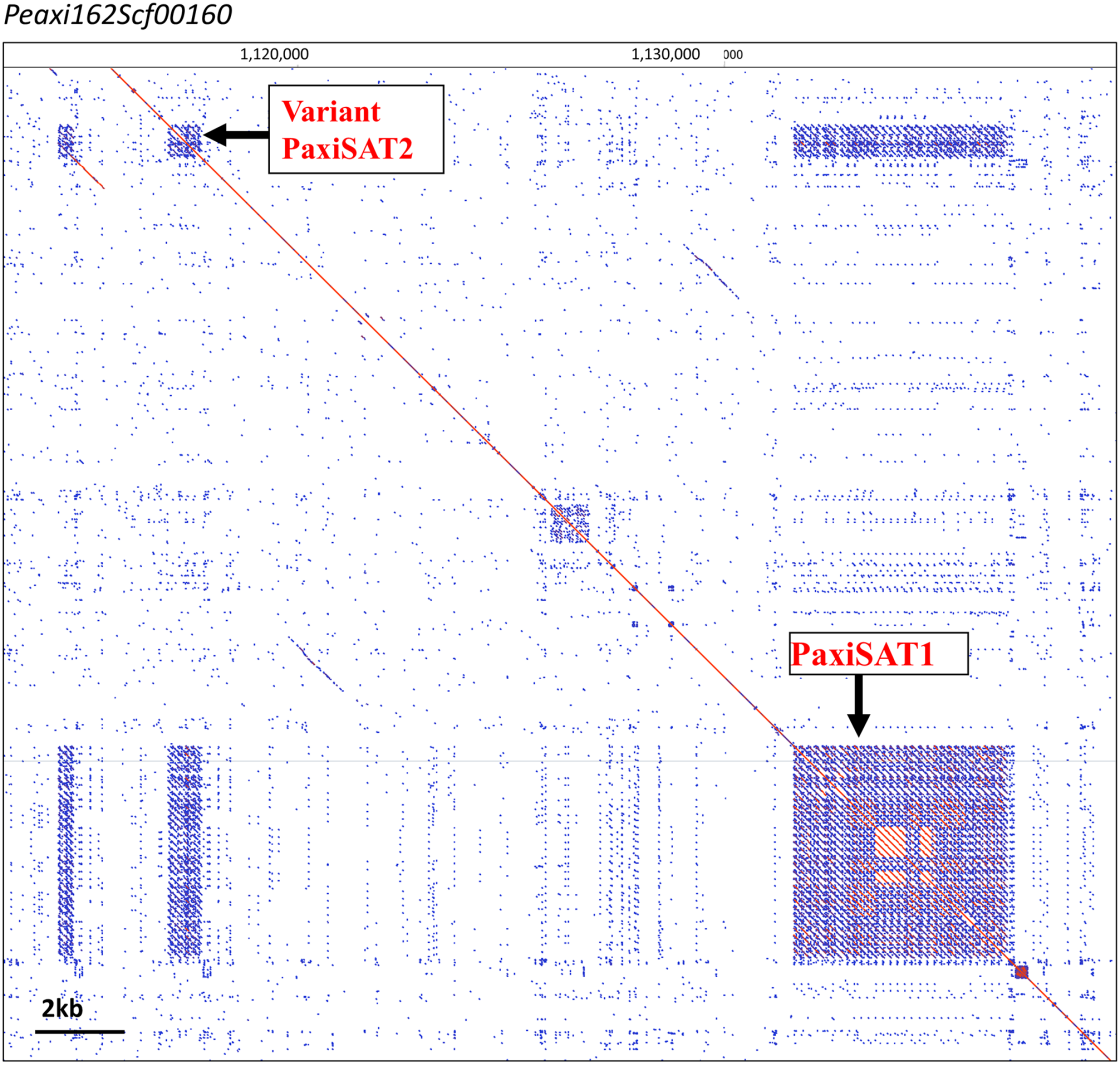
PaxiSAT1 arrays in *Peaxi126Scf00160* nt 1,110,000 to 1,140,000. Self dot blot. The ~5-kb PaxiSAT1 array on the lower right has 29 monomers of 168 bp showing a core of highly homologous sequences (red parallel lines) surrounded by less homologous units (blue lines), while the Variant PaxiSAT2 array consists of five heterogeneous units (for detailed enlargements, see **Supplementary Figure S2**).

**Figure 3:**
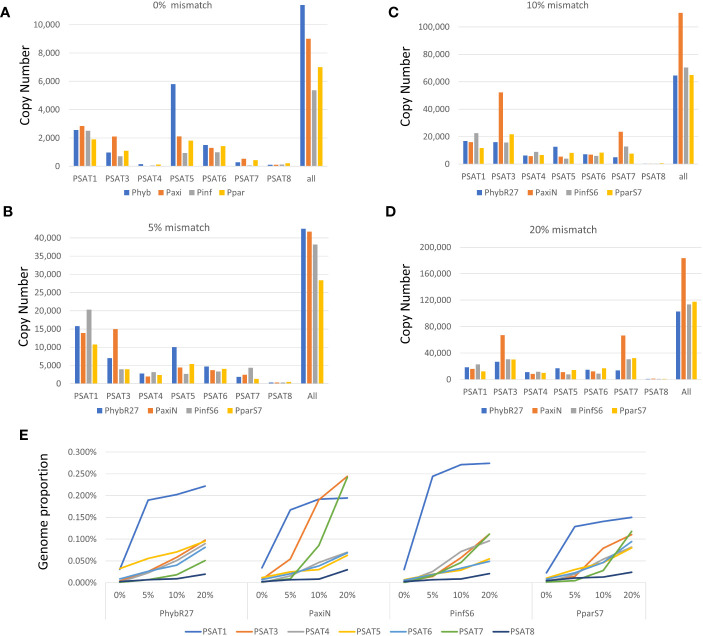
Copy number of repeats. **(A–D)** Short reads that map to consensus sequence were converted to copy numbers using 1.4-Gb *Petunia* genome size. Different amounts of mismatches were allowed when mapping the reads **(A)** 0% mismatch, **(B)** 5% mismatch, **(C)** 10% mismatch, and **(D)** 20% mismatch. **(E)** Genome proportion with different mismatches allowed for each satellite in the four *Petunia* species analyzed. Read data are given in **Supplementary Table S3**.

**Figure 4:**
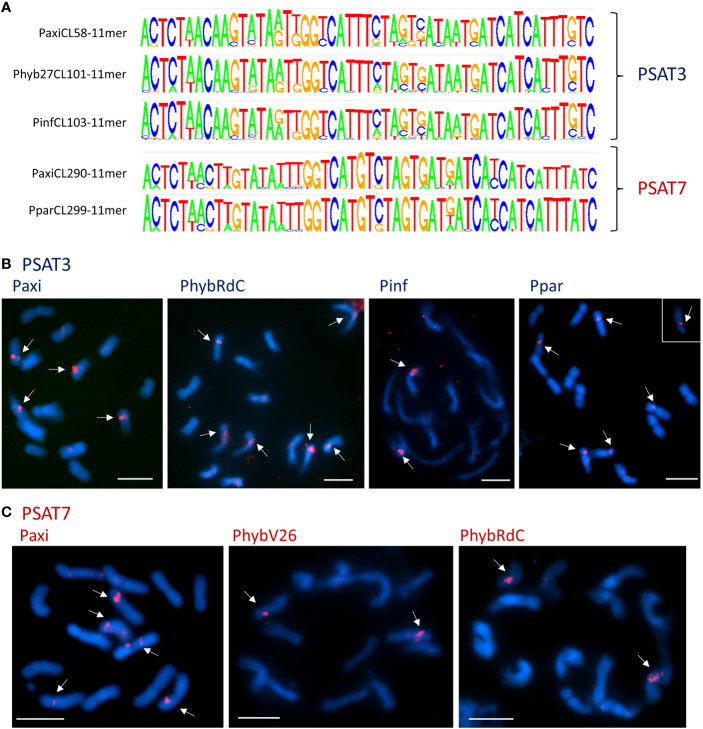
Satellite repeats PSAT3 and variant PSAT7 with a 51-bp monomer. **(A)** Sequence logos of clusters containing the repeats generated by TAREAN k-mer analysis. The consensus sequences between the species are very homologous and show variation at the same positions. Between PSAT3 and PSAT7, there was an 8-nt difference (for sequence alignments, see **Figure S4**). **(B, C)** Fluorescence *in situ* hybridization to metaphase chromosomes (blue with DAPI) of *Petunia axillaris*, *Petunia hybrida* (RdC and V26), *Petunia inflata*, and *Petunia parodii* using the oligonucleotide probes (red signal) for PSAT3 **(B)** and PSAT7 **(C)** showing two to six signals mid-arm or near the centromeres. Bar = 10 µm.

**Figure 5:**
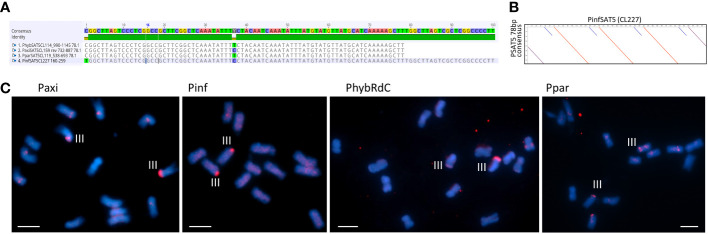
Satellite repeats PSAT5 with a 78- or 100-bp monomer. **(A)** Sequence alignment of the consensus sequence for *Petunia hybrida* R27, *Petunia axillaris N*, *Petunia parodii S7*, and *Petunia inflata* S6. **(B)** Dot plot comparing PinfSAT5 with the 78-bp consensus monomer showing duplication of 28 bp (blue diagonal line). **(C)** Fluorescence *in situ* hybridization to metaphase chromosomes (blue with DAPI) of *P. axillaris*, *P. inflata*, *P. hybrida* (RdC), and *P. parodii* using the oligonucleotide probe PSAT5 (red signal). One pair of strong signals is detected on Chr III and occasionally some minor signals on other chromosomes. Bar = 10 µm.

**Figure 6:**
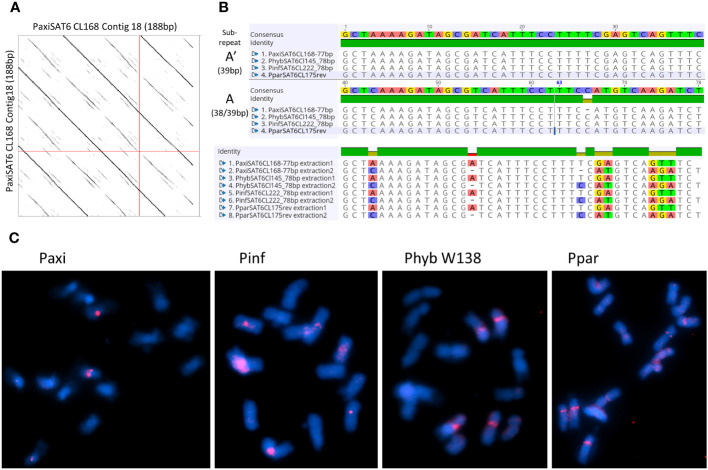
Satellite repeat PSAT6 with 77–78 bp. **(A)** Self dot plot of PaxiSAT6 sequence extracted from Cluster CL168. The 188-bp sequence shows four full repeat units of 77 bp (dark lines) with the subrepeat structure of two 38-bp units with interrupted identities (broken lines). **(B)** Sequence alignments of the consensus sequence for *Petunia axillaris N*, *Petunia hybrida* R27, *Petunia inflata* S6, and *Petunia parodii S7* using sequences extracted from contigs (see **Table S4**). Top shows the alignment of the full 77/78-bp monomer, and bottom shows the alignment of subrepeats A and A′ identifying eight nucleotide positions with alternate bases. **(C)** Fluorescence *in situ* hybridization to metaphase chromosomes (cyan with DAPI) of *P. axillaris*, *P. inflata*, *P. hybrida* (W138), and *P. parodii* using the oligonucleotide probe PSAT6 (red signal). Two to six signals adjacent to the centromeres are visible. Bar = 10 µm.

In the *P. hybrida* R27 (*PhybR27*) reads, graph-based clustering with the program RepeatExplorer and tandem repeat analysis (TAREAN) (Novák et al., 2013; Novák et al., 2017) identified abundant candidate tandem repeats (or putative satellite sequences) by k-mer analysis and characteristic cluster graphs with star- or donut-like forms (**Figure 1A**; **Supplementary Figure S1**). In total, after grouping similar sequences, seven candidate satellite repeat families, most AT-rich, were identified in *Phyb*R27 raw reads (**Table 1**; **Supplementary Tables S2**, **S4**), some being variants of each other (see below). Subsequently, the repeats were identified in the raw reads of *P. axillaris* (*PaxiN*), *P. inflata* (*PinfS6*), the ancestors of the hybrid petunia, and the sister species *P. parodii* (*ParS7*) either as clusters in the TAREAN output or by searching the extracted contigs from RepeatExplorer (**Supplementary Tables S2**, **S5**–**S7**). Each tandem repeat designated PSAT1, PSAT3, PSAT4, PSAT5, PSAT6, PSAT7, and PSAT8 had a characteristic species and overall consensus sequence, monomer length, GC content, and genome proportion and chromosomal organization (**Table 1**, **Figures 1**–**6**; see below). Other bioinformatics approaches were also used to identify tandem repeat motifs, and visualization of dot plots using selected parts of scaffolds from the genome assemblies of *Peaxi162* and *Peinf101* (Bombarely et al., 2016; Schwarzacher et al., 2016) was performed to show tandem arrays by a series of lines of identities parallel to the major diagonal (e.g., **Figure 2**; **Supplementary Figure S2**). These further analyses confirmed the families found by RepeatExplorer and revealed no additional tandem repeat families with motifs more than 50 bp long. We are therefore confident that we exhausted the *P. hybrida* satellite repeatome and captured the major tandem repeat types and families.

**Table 1:** Characteristics of the seven tandem repeat families identified in *Petunia hybrida* (PhybR27), *Petunia axillaris* (PaxiN), *Petunia inflata* (PinfS6), and *Petunia parodii* (PparS7).

Types	Monomer length (bp)	GC content				Genome proportions of clusters containing the satellite repeat				Chromosomal location determined by FISH	Copies in assemblies	
		PhybR27	PaxiN	PinfS6	PparS7	PhybR27	PaxiN	PinfS6	PparS7		PaxiN	PinfS6
PSAT1	168	32.7%	32.7%	33.9%	32.7%	0.190	0.200	0.270	0.150	Telomere associated on all chr except for short arms of Chrs II and III	1,016	1,013
											Arrays size: 5–10 kb	
PSAT3	51	29.4%	29.4%	31.4%	29.4%	0.150	0.290	0.170	0.190	Near the centromere or mid-arm on Chrs II or III and two metacentric chromosomes	4,252	4,316
											Arrays size 3–15 kb	
PSAT4	113	36.3%	35.4%	34.5%	36.3%	0.110	0.092	0.120	0.091	Weak and dispersed signals	8,622	10,007
											Arrays size 1 kb	
PSAT5	78/100	38.5%	39.7%	44.0%	38.5%	0.110	0.130	0.057	0.190	Telomere associated at the end of the short arm of Chr III	45	10
											Arrays size 1 kb	
PSAT6	77–78	39.7%	39.0%	39.7%	39.7%	0.082	0.120	0.060	0.089	Adjacent to the centromere on 2–6 chromosomes (1–3 pairs)	9,595	8,388
											Arrays size 1–2 kb	
PSAT7	51	31.4%	31.4%	31.4%	31.4%	0.021	0.026	not applicable (same as PSAT3)	0.014	Similar to PSAT3, but less signal visible	9,082	5,411
											Arrays size 1–20 kb	
PSAT8	292–298	55.6%	56.2%	55.1%	55.4%	0.011	0.008	0.010	0.017	No signal detected	599	467
											Arrays size 0.1–1 kb	
Total						0.674	0.858	0.687	0.741			

Identified monomer lengths, GC content, and genome proportions within RepeatExplorer clusters and array lengths found in whole genome assemblies of *P. axillaris* v1.6.2 (Peaxi162) and *P. inflata* v1.0.1 (Peinf101).

FISH, fluorescence in situ hybridization.

### 3.2 Abundance and variation of tandem (satellite) repeats in *Petunia* genomes

Genome proportions and copy numbers as identified by RepeatExplorer and TAREAN (**Table S2**) as well as mapping reads to the monomer consensus sequences (**Table S3**) showed that each was abundant with 0.02%–0.2% of the genome and up to several thousand perfect and degenerate copies, with evolutionary differences between repeats and genomes (**Table 1**; **Figure 3**). Copies of all tandem repeat motifs were found in the two genome assemblies of Bombarely et al. (2016), although collapsed by the assembly algorithms, sometimes being placed at ends of scaffolds (**Figure 2**; **Supplementary Figure S2**). In the *P. axillaris* chromosome-level assembly (*Peax403*), we also found tandem repeats underrepresented compared to the proportion of the raw reads being collapsed in assembly or possibly lost in the DNA preparation procedures, although longer arrays were identified (**Supplementary Table S8**).

The genome sizes of the two parental and the hybrid-origin *Petunia* species are similar (1.4 Gb, all 2n = 2x = 14, Bombarely et al., 2016). The expectation would be stochastic processes that lead to differences in abundance in the parental species (separation 30 Mya). *P. hybrida* has a complex history that involved initial hybridization, followed by crosses many times involving different species, hence making it more similar to an introgression line, and not an amphipolyploid (Bombarely et al., 2016 **Supplementary Note 6**); in the gene space of *P. hybrida* R27, approximately 50% genes that could be attributed are thought to be derived from *P. axillaris*, only 5% from *P. inflata*, and at least 16% from other species. Hence, we would not expect an average coverage of *P. inflata* and *P. axillaris* repeats in *P. hybrida*. These three had a similar total genome proportion and copy number of repeats (**Figure 3**; **Supplementary Table S3**), while *P. parodii* S7 had some 30% fewer tandem repeats.

The species consensus sequences of all repeats showed high similarities (**Figures 1**, **4**–**6**; **Supplementary Figure S6**; **Supplementary Data 3**), but there was substantial variation within each sequence: using 0% mismatch, only a few copies were found for each repeat (**Figure 3A**), but the number was much higher with reduced stringency (**Figures 3B–D**; **Supplementary Table S3**). *In situ* hybridization was used to find the location of each repeat on metaphase chromosomes. Except for the least abundant sequence PSAT8 and the more dispersed PSAT4 (where no or only weak hybridization signal was observed), the chromosomal study showed the motifs were present as arrays at multiple discrete loci (**Figures 1E**, **4B, C**, **5C**, **6C**) and in *P. axillaris* corresponding to the distribution as found in the *Peax403* chromosome assembly (see **Table S8**).

### 3.3 Sequence and chromosomal organization of tandem (satellite) repeats

#### 3.3.1 PSAT1

PSAT1 is an AT-rich satellite DNA, represented by 0.2% of the genome, a consensus monomer length of 168 bp, and a GC content of 32.7% (**Figure 1**; **Supplementary Figure S3**; **Tables 1**; **S2**, **S4**-**S7**). The TAREAN and RepeatExplorer clustering graphs for PSAT1 show condensed star-like shapes (**Figure 1A**), and the consensus sequences for *P. hybrida* R27, *P. axillaris*, *P. inflata*, and *P. parodii* are highly conserved with only five bases out of the 168 bp showing variation (**Figure 1B**). Sequence variation within the species is also small, as shown by sequence logos generated by mapping the PaxiN short reads to the PaxiPSAT1 consensus sequence using stringent conditions (**Figure 1C**) or by the TAREAN k-mer analysis (**Supplementary Figure S3A**). PSAT1 contains several direct and indirect short repeats (**Figure 1D**) and shows identity to repetitive DNA Clone pCAS78 of (Shepherd et al. (1990); see **Supplementary Figures S3B, C**). The genomic clone PaxiSAT1 contains a 155-bp part of the monomer (**Table S5**).

*In situ* experiments showed that PSAT1 sequences were at telomere-associated regions, with two dots on most chromosome ends in the four *Petunia* species (**Figure 1E**). PSAT1 signal was not detected on the short arms of Chrs II and III; Chr II was identified by the 5S rDNA arrays located close to the centromere on the short arm. This corresponds to the absence of PSAT1 at both ends of Chromosome 2 and one end of Chromosome 3 of the *Peax403* chromosome assembly of Kuhlemeier and Cannarozzi, 2022. All other chromosomes had PSAT1 arrays of varying lengths at the very end of the final 100-kb region (**Table S8**). We conclude that PSAT1 is a TAS.

PSAT1 sequences were also found in large arrays of several kb in the *Peaxi162* and *Peinf101* assemblies of Bombarely et al. (2016) and marked the end of several scaffolds and shows interruption of satellite arrays with LTR retroelements (for examples, see **Figure 2**; **Supplementary Figure S2**), and the repeat was earlier noted, but not further analyzed, in scaffold *Peaxi162Scf00160* (Schwarzacher et al., 2016 **Supplementary Note 2** to Bombarely et al., 2016). Within the scaffold, there is a 5-kb PaxiSAT1 array that contains 29 monomers (**Figure 2**) with six highly homologous sequences in the center as well as more diverged units particularly toward the ends of the array (**Supplementary Figure S3D**). Another small array of 779 bp, approximately 15 kb away, with five more divergent units, Variant PaxiSAT2, is also present on *Peaxi162Scf00160*, showing 75% identity to the true PaxiSAT1 (**Figure 2**; **Supplementary Figure S3E**). Scaffold *Peaxi162Scf00160* is located at the end of Chromosome 5 in the (2022) *Peax403* of Kuhlemeier and Cannarozzi (**Supplementary Figure S2**). The presence of only a few highly homologous monomers and many more degenerate units in this array is supported by the high number of approximately 420,000 raw reads in *PaxiN* when reducing the stringency in our map to reference analysis compared to only 57,000 copies when using stringent conditions (**Supplementary Table S3**); the raw reads of *PinfS6*, *PhybR27*, and *PparS7* similarly show a 5–6x increase of copies when considering 20% mismatch. The low number of approximately 1,000 copies in the *Peinf101* and *Peaxi162* assemblies is likely to represent a collapse of monomers in the assemblies, and an estimate of more than 10,000–20,000 copies of PSAT1 per genome is more likely as found in the raw reads *PhybR27*, *PaxiN*, and *PinfS6* and a lower content in *PparS7* (**Figure 3**; **Supplementary Table S3**).

#### 3.3.2 PSAT3 and variant PSAT7

Tandem repeat PSAT3 has a monomer of 51 bp and is 30% GC rich; it is abundantly represented in the assemblies and raw reads with 0.15%–0.2% genome proportion (**Table 1**; **Figure 4**). It showed variable sizes of arrays between 3 kb and 15 kb within small scaffolds, and different copy numbers were present in each species showing many more degenerate monomers than those with highly conserved monomers (**Supplementary Table S3**). In TAREAN and RepeatExplorer, the repeat graphs showed a solid star-like shape with one to more arms (**Supplementary Figure S1A**).

The consensus PSAT3 repeat showed little sequence variation within and between species as shown by the sequence logos and comparing species consensus sequences (**Figure 4A**; **Supplementary Figure S4A**). PSAT3 has a very high identity to the same length PSAT7 with only 8 of the highly conserved 51 bp, 15.7% (**Supplementary Figure S4B**). The two repeats can be viewed as variants, although they were found in different RepeatExplorer clusters in the raw reads *PhybR27*, *PaxiN*, and *PparS7*, albeit PSAT7 was approximately 10× less abundant (see **Table 1**; **Figure 3**; **Supplementary Tables S4**, **S5**, **S7**); in *PinfS6*, PSAT3 and PSAT7 were in the same cluster (**Table S6**).

On chromosomes, using the 51-bp consensus sequences of PSAT3 and PSAT7 as oligo FISH probes, cross-hybridization of the two probes is expected to occur at the stringency of approximately 80% used for the experiment, and indeed, the signal distribution is similar but with PSAT7 showing fewer sites (**Figures 4B, C**; **Supplementary Figures S4C, D**). With the PSAT3 probe, a strong signal was found on two pairs of *P. axillaris* N chromosomes, the mid-arm of Chrs II or III, and near the centromere of a medium-sized metacentric chromosome, as well as a weak signal mid-arm of another medium metacentric chromosome (**Figure 4B**); this corresponds to the presence of the PSAT3 repeat in the *Peax403* assembly. Four to six signals were detected with the PSAT3 probe in *P. hybrida* accessions and *P. parodii* S7 near the centromeres to mid-arm (**Figure 4B**; **Supplementary Figure S4C**), while PSAT7 only detected one pair of signals (**Figure 4C**; **Supplementary Figure S4D**). In *P. inflata* S6, more sites were detected with the PSAT7 probe than the PSAT3 probe, indicating a better fit of the PSAT7 oligo probe to the repeat monomer in this species.

#### 3.3.3 PSAT5

Although the TAREAN reports did not identify PSAT5 as a putative satellite sequence other than in *P. inflata* (**Supplementary Table S2**), the cluster graphs containing the repeat sequence showed characteristics of satellites (**Supplementary Figure S1C**). A 78-bp monomer was found in the extracted contigs of *PaxiN*, *Phyb27*, and *PparS7* clusters, while in *PinfS6*, a 100-bp repeat unit with an internal duplication was present (**Tables S4**-**S7**; **Figures 5A, B**); PSAT5 is approximately 40% GC rich (slightly higher in *P. inflata*). PSAT5 was poorly represented within *Peinf101* and *Peaxi162* assemblies (as small 1-kb arrays; **Table 1**), although copies were relatively abundant in all raw reads (0.08% genome proportion) and overall showed high sequence conservation, as 20% mismatch in the map to reference analysis increases the copy numbers less than for other repeats (**Figure 3**; **Supplementary Table S3**). In the *Peax403* assembly, similarly, only a few copies were dispersed throughout the genome, except near the end of Chromosome 3 where a large array was found (**Supplementary Table S8**). *In situ* hybridization showed a few weak signals at various positions or dispersed along the chromosomes and a very strong signal at the end of the short arm of Chr III in all *Petunia* species (**Figure 5C**; **Supplementary Figure S5A**).

#### 3.3.4 PSAT6

PSAT6 has a 78-bp (77 bp for *P. axillaris*) TAREAN consensus sequence with approximately 40% GC content; it can be subdivided into two similar subrepeats (A and A′) of 38 and 39 bp (**Figures 6A, B**). Arrays in contigs extracted from RepeatExplorer clusters from *Phyb27*, *PaxiN*, *Pinf*, and *Ppar* (**Supplementary Tables S4**-**S7**) show variable numbers of subrepeats of either 38 bp or 39 bp, arranged alternatively or with some degenerations and some 65%–70% identity to each other; many more copies were also found when allowing 20% mismatch in the map to reference analysis (**Figure 3**; **Supplementary Table S3**). Abundant PSAT6 sequences have been found in the two assemblies *Peaxi162* and *Peinf101*; arrays found were between 1 and 2 kb, with additional dispersed units. Notably higher copy numbers in *PaxiN* and *PhybR27* reads than in the other two species were present (**Table 1**). PSAT6 using the subrepeat A as an oligo probe for FISH, signals of variable strength were visible adjacent to the centromeres of two to six chromosomes (**Figure 6C**; **Supplementary S5B**).

#### 3.3.5 PSAT4 and PSAT8

These two repeats were identified as putative satellite sequences in the TAREAN reports (**Supplementary Table S2**), but fluorescence *in situ* hybridization to chromosomes showed weak and dispersed or no signal (**Supplementary Figure S6**) despite PSAT4 being approximately 0.1% of the genomes and PSAT8 approximately 0.02% (**Table 1**). PSAT4 is a 113-bp monomer with a 35% GC content, while PSAT8 has an almost 300-bp monomer and rather higher GC content at 55% (**Supplementary Figures S6A, B**; **Tables S4**-**S7**). Map to reference analysis shows that PSAT4 is very variable, finding very few (less than 1,000) copies with 0% mismatch to the consensus, but many (up to 150,000) copies allowing 10% or 20% mismatch (**Figure 3**; **Supplementary Table S3**). Within the assemblies, small arrays were found approximately 10 times more for PSAT4 than PSAT8 (**Table 1**). Within the *Peaxi403* assembly, both repeats were found, but they are dispersed over all chromosomes and mainly in copies of less than 6 in one region (**Table S8**).

## 4 Discussion

### 4.1 Copy numbers of tandemly repeated elements

Our analysis of repetitive elements from genomes of four *Petunia* species (*P. axillaris*, *P. inflata*, *P. hybrida*, and *P. parodii*) identified seven tandem repeat or satellite families (PSAT1, PSAT3, PSAT4, PSAT5, PSAT6, PSAT7, and PSAT8) not found in the Solanaceae crown group genera (*Nicotiana*, *Solanum*, and *Capsicum*) and showed their abundance and diversity using complementary tools (graph-based repeat clustering using unassembled raw reads, k-mer analysis, read mapping, sequence assemblies, *in situ* hybridization, and chromosome studies). In total, all repetitive elements represented 64%–68% of the *Petunia* genomes, consistent with the measurements reported by Bombarely et al. (2016) in *P. axillaris* N and *P. inflata* S6, and was composed of abundant DNA transposons, LTR retroelements, and retrotransposons including pararetroviruses, as seen in other Solanaceae species (Hansen and Heslop-Harrison, 2004; Staginnus and Richert-Pöggeler, 2006; Staginnus et al., 2007; Richert-Pöggeler and Schwarzacher, 2009). A very low proportion of the genome, approximately 0.8% (10 Mb of the c. 1,400 Mb genomes, excluding the rDNA and short tandem repeats or simple sequence repeats), was represented by a limited number of tandemly repeated (satellite DNA) families, each less than 0.3% of the genome (**Table 1**). In contrast, within the Solanaceae crown x = 12 clade, many repeat families in *Solanum* (Tang et al., 2014), *Nicotiana* (Hemleben et al., 2007), and *Capsicum* (Zhou et al., 2019; de Assis et al., 2023) have been found each at similar or more often higher abundance and a total higher satellite genome proportion. Within the *Solanum* potato and tomato clade, satellite DNAs typically total 1%–8% of the genome (Gaiero et al., 2019), and the CL14 satellite repeat (Torres et al., 2011) alone represents up to 7% of different tomato clade genomes. Within other plant families, satellite repeats have been reported at various frequencies, mainly between 2% and 10% genome proportion, e.g., 6.8% in *Beta vulgaris* (750 Mb, Zakrzewski et al., 2010), 3.3% *Arachis glandulifera* (1315 Mb, Samoluk et al., 2019), 2% in *Avena* (3,900 Mb, Liu et al., 2019), and 3% in *Cenchrus ciliata* (1,500 Mb, Rathore et al., 2022). *Arabidopsis thaliana* (176 Mb) includes 4% of the *AtCon* tandemly repeated centromeric sequence (Heslop-Harrison et al., 1999), although species with no significant satellite sequences are also known, i.e., *Chrysanthemum nankingense* (Zhang et al., 2023).

The low proportion of satellites in *Petunia* is supported by the chromosome images where no positively or negatively stained bands are seen after DAPI staining, except at the 45S rDNA sites sequences, the secondary constrictions, on Chrs II and III (**Figures 1E**, **4B, C**, **5C**, **6C**). In contrast, many other plant species have conspicuous bands, considered heterochromatin and rich in satellite repeats, at centromeric, sub-telomeric, or intercalary positions on chromosomes (e.g., Contento et al., 2005; Hemleben et al., 2007; Moscone et al., 2007; Mlinarec et al., 2019; de Souza et al., 2022). In *Petunia*, as in many other species (Heslop-Harrison and Schwarzacher, 2011), the peri-centromeric regions are characterized by retroelement-related sequences (Richert-Pöggeler and Schwarzacher, 2009; Bombarely et al., 2016). In our analysis, we found no satellite repeat that was prominent at all centromeres or contained centromere-like motifs that would indicate CenH3 binding sites typical for centromere-specific tandem repeats (Heslop-Harrison et al., 1999; Gong et al., 2012; Kirov et al., 2018; Peška et al., 2019). Some repeat families identified here, though, have positions adjacent to the centromeres of a few chromosomes. The most prominent satellite sequence identified, PSAT1, has a repeat unit of 168 bp in the range postulated to wrap around the nucleosomes (Henikoff et al., 2001; Heslop-Harrison and Schwarzacher, 2013), and localization at the ends of all chromosomes (**Figure 1**) can be viewed as a TAS.

### 4.2 Chromosomal locations of satellite sequences

Of the seven repeat families, five showed distinct and characteristic signals on chromosomes (**Figures 1E**, **4B, C**, **5C**, **6C**) with signal strength matching genome proportion and arrays found in available whole genome sequence assemblies (**Table 1**). For those two satellites with no distinct FISH signal, we assume a rather dispersed distribution, and individual arrays are not large enough to allow the probe to show visible hybridization signals. PSAT1 showed a telomeric to subtelomeric location in all four *Petunia* species analyzed, with distinct double dots at the ends of most chromosome arms (**Figure 1E**). Some chromosome ends in the *Peaxi403* assembly of Kuhlemeier and Cannarozzi, 2022 showed the true telomeric sequence of (TTTAGGG) distal to the PSAT1 arrays. Together with the chromosomal location of PSAT1, the results suggest that it is a telomere-associated sequence, although we did not find degenerate telomere sequence motifs as has been reported for *Nicotiana plumbaginifolia*, rye, and wheat (Chen et al., 1997; Contento et al., 2005) nor a more G or more C-rich strand in the highly AT-rich sequence with multiple As or Ts (**Figures 1B, C**). We found only one satellite sequence family associated with the telomere, while in potato two sequences, the more homogenous CL14 and the less conserved CL34 family were at the chromosome ends, sometimes together and sometimes separately, and for two ends missing (Torres et al., 2011), and in *Capsicum* species, the subtelomeric satellite repeats CDR-1 and CDR-2 are present at different amounts and distribution (de Assis et al., 2023). In Triticeae, the subtelomeric heterochromatin contains the more widely distributed pSc119.2 telomere-associated sequence that is also found interstitially, but in *Secale*, additionally, highly repetitive sub-genus-specific satellite families are present between pSc119.2 and the telomere (see Vershinin and Heslop-Harrison, 1998; Contento et al., 2005). PSAT1 sequences were missing from the short 45S rDNA bearing arms of Chr II and III. This is similar to the situation of *Tanacetum cinerariifolium* (Asteraceae, Mlinarec et al., 2019) where two telomere-associated satellite sequence families are present, but not at the ends of chromosome arms with distal 45S rDNA. In rye, the TAS sequences are, however, present at the short arm of Chromosome 1R bearing the single nucleolus organizer region. Interestingly, PSAT5 has large arrays and a strong signal on the short arm of Chr III but shows no sequence similarity with PSAT1 and would not be assumed to be a telomere-associated sequence. The other PSAT families showed centromere adjacent or intercalary signals with variable strength on one to three chromosome pairs and with differences between species and accessions, but none could be classified as a universal *Petunia* centromere satellite repeat as described in other Solanaceae (Gong et al., 2012).

### 4.3 Diversity and evolution of repetitive elements

Many studies have shown that satellite sequences are a rapidly evolving component of the genome, even if only a small component; they are valuable for identifying species and chromosome relationships (Heslop-Harrison and Schwarzacher, 2011) and for exploiting knowledge of sequences that are restricted to closely related species, distributed more widely within a genus or tribe or that show distinctive chromosomal distribution. Studies cover a wide range of families in the angiosperms (see Hemleben et al., 2007) and gymnosperms (Heitkam et al., 2021). The seven tandem repeat families here (PSAT1, PSAT3, PSAT4, PSAT5, PSAT6, PSAT7, and PSAT8) were present in all four genomes analyzed, *P. axillaris*, *P. inflata*, *P. hybrida*, and *P. parodii*. These repeat families showed sequence similarities and organization, indicating their presence in the common ancestor but, except for PSAT3 and PSAT7, were unrelated in motif length or sequence. *Petunia* species separated from the common Solanaceae ancestor approximately 30 Mya ago (Wang et al., 2008; Bombarely et al., 2016), and no sequences found here showed identities to satellites identified in other Solanaceae. *Petunia* species themselves have a relatively recent origin of speciation, probably only 0.5 Mya (Gerats and Strommer, 2009; Stehmann et al., 2009), and the species we have analyzed have weak boundaries mainly maintained through geographical separation or different pollination systems, but little genetic difference. They undergo frequent hybridization and back-crossing events as evidenced by *P. hybrida* in nature and breeding programs, allowing frequent exchange of sequences. Not surprisingly, we found no species-specific retroelements (Richert-Pöggeler and Schwarzacher, 2009) and no species-specific satellite repeats or variants and limited variation in their abundance or chromosomal distribution (this study), unlike the sister genus *Nicotiana* (Gazdová et al., 1995; Lim et al., 2006) or *Solanum* (Tang et al., 2014; Gaiero et al., 2019). There seems to be a variable relationship between repetitive DNA, dispersed and tandemly repeated satellite DNAs, and genome size as different rates of evolution and strong phylogenetic signal of major repeat sequences, and up and down genome sizing can occur in diploid species (McCann et al., 2020; Chase et al., 2023). Genomic changes are regularly seen in hybrid species (Alix et al., 2017). Although *P. hybrida* is probably only 200 years old (Bombarely et al., 2016), and genomic changes happen over thousands of years in established hybrid species cannot be expected, although recent studies in resynthesizing polyploids and hybrid species have shown rapid adaptations and sequence losses or gains in *Brassica* tetraploids (Gaeta et al., 2007) or triticale (wheat x rye; Ma and Gustafson, 2008). Within *Petunia*, the consensus sequences of the satellite repeats were remarkably similar between the species (**Figures 1**, **4**–**6**), showing little evolutionary differences between the genomes, although the *P. inflata* genome could be separated by having a longer repeat unit for PSAT6 due to an internal duplication, and PSAT7, the variant to PSAT3 found in *P. hybrida*, *P. axillaris*, and *P. parodii*, was not identified separately. It was, thus, not possible to identify if the *P. hybrida* genome had sequences inherited from *P. axillaris* or *P. inflata*. All repeats showed low copies of perfect repeat units and many more degenerate copies. This was evident in the contigs extracted from the clusters (**Supplementary Data Tables S4**-**S7**). Notably, analysis of sequence variation by mapping reads at different stringencies to the consensus (**Figure 3**) showed that repeats had different characteristics with respect to homogenization or diversification. PSAT1 and PSAT5 showed only a small increase in genome proportions at all stringencies, reflected also in the sequence logo and the organization found in the assemblies (**Figures 1**, **2**, **5**). In contrast, PSAT3 was much more variable in sequence, particularly in *P. axillaris* where it was the most frequent satellite, with many more reads mapping to the consensus at low (15%–20% mismatch) than at higher stringency. The differences may reflect mechanisms homogenizing the arrays (cf. gene conversion) being more active with some PSAT sequences than others or within some genomes (such as was postulated for the gene space in *P. axillaris*, Bombarely et al., 2016). The detailed analysis here adds to information about modes of satellite amplification, and we suggest that the mechanisms—and hence consequences for monomer homogeneity and nature of tandem repeat arrays—may differ between satellite origins, their sequence, their evolutionary ages, chromosomal location, and species. Replication slippage and uneven crossing-over can change copy number, as can amplification within retroelements, while sequence homogenization can lead to divergence of sequence motifs in different species The deviation of sequence copies can provide evidence for the nature of amplification of particular variants from a common ancestor, the library hypothesis (Mestrović et al., 1998; Kuhn et al., 2010), although there is little evidence in the *Petunia* species for amplification of such variants, nor of different ages of satellite DNA monomers as found in Bovidae (Escudeiro et al., 2019). Similar to oedipodine grasshoppers (Camacho et al., 2022), the *Petunia* satellites, however, show some point mutations combined with amplification or loss in the recently evolved hybrid species.

## 5 Conclusions

*P. hybrida*, as a diploid, 2x = 14 hybrid species, has an unusual genome constitution where the contributing ancestral genomes *P. axillaris* and *P. inflata* have contributed unequal amounts to the hybrid genome due to frequent backcrosses. Tandem satellite repeats, a smaller part of the *Petunia* genome compared to the Solanaceae x = 12 clade of *Solanum*, *Nicotiana*, and *Capsicum*, have distinct chromosomal and sequence organization maintained in *P. hybrida* with only small changes in copy number, and limited but satellite-specific homogenization rates and events. The satellite sequences identified are unique to the petunia genomes, and the seven families identified were present in all the genomes investigated but were not found in other Solanaceae species, supporting the unique makeup of petunia chromosomes and the unique position of *Petunia* (and its sister genus *Calibrachoa*) within the Solanaceae having split from the x = 12 crown clade species 30 Mya (Bombarely et al., 2016). Although satellites have small genomic proportions, with distinct chromosomal positions, such as PSAT1 as a telomere-associated sequence, or those associated with the centromeres, we can suggest that they likely play a role in the maintenance of nuclear structure at metaphase and interphase and during chromosome pairing at meiosis (Hemleben et al., 2007; Heslop-Harrison and Schwarzacher, 2011; Sepsi and Schwarzacher, 2020). Large and smaller arrays of uniform and degenerate repeats of maintained monomer length of 50–170 bp, as seen here in the different satellite families, have been described previously as important for chromatin packaging and chromosome stability (Henikoff et al., 2001; Heslop-Harrison and Schwarzacher, 2013). Overall, these findings provide insights into the recent evolution of the *P. hybrida* genome and its satellitome, shedding light on the conservation, variation, and localization patterns of tandemly repeated sequences in different *Petunia* species. The study contributes to the fundamental understanding of evolution and genomic changes in one of the components of the most abundant fraction of the genome, including changes associated with hybridization events.

## Data availability statement

The datasets presented in this study can be found in online repositories. The names of the repository/repositories and accession number(s) can be found below: https://www.ncbi.nlm.nih.gov/genbank/, OQ579142 https://www.ncbi.nlm.nih.gov/genbank/, OQ579144 https://www.ncbi.nlm.nih.gov/genbank/, OQ579145 https://www.ncbi.nlm.nih.gov/genbank/, OQ579146 https://www.ncbi.nlm.nih.gov/genbank/, OQ579147 https://www.ncbi.nlm.nih.gov/genbank/, OQ579148 https://www.ncbi.nlm.nih.gov/genbank/, OQ579149 https://www.ncbi.nlm.nih.gov/genbank/, OQ676819 https://www.ncbi.nlm.nih.gov/, SRR22797038. https://figshare.com/, s/1ce1559d6e7474984a14. Additional Supplementary Data 1,2 and 3 can be found as file ‘Petunia_tandemly_repeated_DNA_AlisawiRichertPoggelerHeslopHarrisonSchwarzacherAdditionalInformation_DatasetForThisStudy’ in the link https://figshare.com/s/1ce1559d6e7474984a14.

## Author contributions

JH-H, TS, and KR-P conceived the research and supervised the project. OA performed the experiments and analyzed the data together with JH-H, KR-P, and TS. OA and TS wrote the manuscript, and all authors corrected and approved the final version.
