# Supplementary material for: The nature and organization of satellite DNAs in Petunia hybrida, related, and ancestral genomes
Source: Front Plant Sci. 2023 Oct 6;14:1232588. doi: 10.3389/fpls.2023.1232588 (PMC10587573; doi:10.3389/fpls.2023.1232588)
Supplement: Supplementary file 1 [file DataSheet_1.zip › Table S2.docx]

**Alisawi et al. Petunia satellite repeats**

**Supplementary material**

**Table S2:** **TAREAN cluster summary for the analysed satellites repeats (PSATs) in *P. hybrida, P. axillaris, P. inflata and P.parodii*.**

Note that repeated TAREAN runs might give different cluster numbers; clusters are ranked according to genomic abundance and as the clusters are present with very small differences in abundance, clusters are easily ranked differently. Some repeats did not appear in the TAREAN report as putative satellites, but clusters were identified manually, and sequences extracted from contigs (see Suppl. Tables S4 to S7).

| Repeat | R27  Second run | Paxi | Pinf | Ppar |
| --- | --- | --- | --- | --- |
| PSAT1 | (CL80)   \| size \| 957 \| \| --- \| --- \| \| size_real \| 957 \| \| ecount \| 253289 \| \| supercluster \| 33 \| \| annotations_summary \|  \| \| pair_completeness \| 0.99375 \| \| pbs_score \| 0.0 \| \| TR_score \| 0.7073563878570039 \| \| TR_monomer_length \| 168.0 \| \| loop_index \| 0.991640543364681 \| \| satellite_probability \| 0.986367215261502 \| | (CL107)   \| size \| 2743 \| \| --- \| --- \| \| size_real \| 2743 \| \| ecount \| 1619884 \| \| supercluster \| 30 \| \| annotations_summary \|  \| \| pair_completeness \| 0.979076479076479 \| \| pbs_score \| 0.0 \| \| TR_score \| 0.7050730797481204 \| \| TR_monomer_length \| 168.0 \| \| loop_index \| 0.991979584396646 \| \|  \|  \| \| satellite_probability \| 0.992045020040958 \| | (CL63)   \| size \| 3916 \| \| --- \| --- \| \| size_real \| 3916 \| \| ecount \| 3341270 \| \| supercluster \| 29 \| \| annotations_summary \|  \| \| pair_completeness \| 0.990849008642603 \| \| pbs_score \| 0.0 \| \| TR_score \| 0.7058653074353008 \| \| TR_monomer_length \| 168.0 \| \| loop_index \| 0.993105209397344 \| \| satellite_probability \| 0.986367215261502 \| | (CL147)   \| size \| 1762 \| \| --- \| --- \| \| size_real \| 1762 \| \| ecount \| 934228 \| \| supercluster \| 43 \| \| annotations_summary \|  \| \| pair_completeness \| 0.970917225950783 \| \| pbs_score \| 0.0 \| \| TR_score \| 0.7171503397219305 \| \| TR_monomer_length \| 168.0 \| \| loop_index \| 0.939273552780931 \| \| satellite_probability \| 0.911290703863514 \| |
| PSAT3 | (CL101)   \| size \|  \| 729 \| \| --- \| --- \| --- \| \| size_real \|  \| 729 \| \| ecount \|  \| 13783 \| \| supercluster \|  \| 42 \| \| annotations_summary \|  \|  \| \| pair_completeness \|  \| 0.869230769230769 \| \| pbs_score \|  \| 0.0 \| \| TR_score \|  \| 0.4888554405769834 \| \| TR_monomer_length \|  \| 51.0 \| \| loop_index \|  \| 0.839506172839506 \| \| satellite_probability \|  \| 0.0713717197270795 \| | (CL58)   \| size \| 3952 \| \| --- \| --- \| \| size_real \| 3952 \| \| ecount \| 750728 \| \| supercluster \| 13 \| \| annotations_summary \|  \| \| pair_completeness \| 0.899086977414704 \| \| pbs_score \| 0.0 \| \| TR_score \| 0.46753139080118017 \| \| TR_monomer_length \| 51.0 \| \| loop_index \| 0.957742914979757 \| \| satellite_probability \| 0.888792941251299 \| | (CL103)  CL103 has two satellite sequences PaxiSAT3 and PaxiSAT7.   \| size \| 2469 \| \| --- \| --- \| \| size_real \| 2469 \| \| ecount \| 129904 \| \| supercluster \| 15 \| \| annotations_summary \|  \| \| pair_completeness \| 0.874715261958998 \| \| pbs_score \| 0.3158386 \| \| TR_score \| 0.4481865048170966 \| \| TR_monomer_length \| 51.0 \| \| loop_index \| 0.870392871607938 \| \| satellite_probability \| 0.0741261545090387 \| | (CL117)  The cluster was not identified as putative satellite in the TAREAN report of this run, but homologous sequences were identified in contigs of this cluster. |
| PSAT4 | (CL116)   \| size \| 557 \| \| --- \| --- \| \| size_real \| 557 \| \| ecount \| 12675 \| \| supercluster \| 52 \| \| annotations_summary \|  \| \| pair_completeness \| 0.735202492211838 \| \| pbs_score \| 0.0 \| \| TR_score \| 0.6033693658397592 \| \| TR_monomer_length \| 113.0 \| \| loop_index \| 0.727109515260323 \| \| satellite_probability \| 0.0204127423022226 \| | (CL202)   \| size \| 1246 \| \| --- \| --- \| \| size_real \| 1246 \| \| ecount \| 53978 \| \| supercluster \| 49 \| \| annotations_summary \|  \| \| pair_completeness \| 0.665775401069519 \| \| pbs_score \| 0.0 \| \| TR_score \| 0.5524964561348243 \| \| TR_monomer_length \| 113.0 \| \| loop_index \| 0.796950240770466 \| \| satellite_probability \| 0.0369466911255025 \| | (CL156)   \| size \| 1691 \| \| --- \| --- \| \| size_real \| 1691 \| \| ecount \| 135482 \| \| supercluster \| 50 \| \| annotations_summary \|  \| \| pair_completeness \| 0.74870734229576 \| \| pbs_score \| 0.0 \| \| TR_score \| 0.6359884376980506 \| \| TR_monomer_length \| 113.0 \| \| loop_index \| 0.846244825547014 \| \| satellite_probability \| 0.0490011209753026 \| \|  \|  \| | (CL173)   \| size \| 1085 \| \| --- \| --- \| \| size_real \| 1085 \| \| ecount \| 46956 \| \| supercluster \| 61 \| \| annotations_summary \|  \| \| pair_completeness \| 0.755663430420712 \| \| pbs_score \| 0.0741274 \| \| TR_score \| 0.5553407370771698 \| \| TR_monomer_length \| 113.0 \| \| loop_index \| 0.749308755760369 \| \| satellite_probability \| 0.027974219694915 \| |
| PSAT5 | (CL114)  The cluster was not identified as putative satellite in the TAREAN report of this run, but homologous sequences were identified in contigs of this cluster. | (CL159)  The cluster was not identified as putative satellite in the TAREAN report of this run, but homologous sequences were identified in contigs of this cluster. | ( CL 227)  .Repeat unit is only 22bp, but longer monomers found in contigs   \| size \| 812 \| \| --- \| --- \| \| size_real \| 812 \| \| ecount \| 50858 \| \| supercluster \| 13 \| \| annotations_summary \|  \| \| pair_completeness \| 0.845454545454545 \| \| pbs_score \| 1.252021 \| \| TR_score \| 0.48120005006168315 \| \| TR_monomer_length \| 22.0 \| \| loop_index \| 0.966748768472906 \| \| satellite_probability \| 0.738793884224469 \| \| consensus \| TCGGCTTAGTCGCTCGGCCGCT \| | (CL119)  The cluster was not identified as putative satellite in the TAREAN report of this run, but homologous sequences were identified in contigs of this cluster. |
| PSAT6 | (CL145)  The cluster was not identified as putative satellite in the TAREAN report of this run, but homologous sequences were identified in contigs of this cluster. | (CL168)  The cluster was not identified as putative satellite in the TAREAN report of this run, but homologous sequences were identified in contigs of this cluster. | (CL222)   \| size \| 859 \| \| --- \| --- \| \| size_real \| 859 \| \| ecount \| 54479 \| \| supercluster \| 11 \| \| annotations_summary \|  \| \| pair_completeness \| 0.721442885771543 \| \| pbs_score \| 0.0 \| \| TR_score \| 0.3826169857789783 \| \| TR_monomer_length \| 39.0 \| \| loop_index \| 0.720605355064028 \| \| satellite_probability \| 0.0173810038896927 \| | (CL175)  The cluster was not identified as putative satellite in the TAREAN report of this run, but homologous sequences were identified in contigs of this cluster. |
| PSAT7 | (CL302)   \| size \| 104 \| \| --- \| --- \| \| size_real \| 104 \| \| ecount \| 758 \| \| supercluster \| 224 \| \|  \|  \| \| pair_completeness \| 0.925925925925926 \| \| pbs_score \| 0.0 \| \| TR_score \| 0.5179228334331423 \| \| TR_monomer_length \| 51.0 \| \| loop_index \| 0.769230769230769 \| \| satellite_probability \| 0.0616570494805888 \| | (CL290)   \| size \| 359 \| \| --- \| --- \| \| size_real \| 359 \| \| ecount \| 13541 \| \| supercluster \| 108 \| \| annotations_summary \|  \| \| pair_completeness \| 0.850515463917526 \| \| pbs_score \| 0.0 \| \| TR_score \| 0.5207578097910034 \| \| TR_monomer_length \| 51.0 \| \| loop_index \| 0.949860724233983 \| \| satellite_probability \| 0.727332981713552 \| | (CL103)  CL103 has two satellite sequences PaxiSAT3 and PaxiSAT7 | (CL299)   \| size \| 165 \| \| --- \| --- \| \| size_real \| 165 \| \| ecount \| 2163 \| \| supercluster \| 180 \| \| annotations_summary \|  \| \| pair_completeness \| 0.833333333333333 \| \| pbs_score \| 0.0 \| \| TR_score \| 0.5253771692683352 \| \| TR_monomer_length \| 51.0 \| \| loop_index \| 0.921212121212121 \| \| satellite_probability \| 0.371632470172109 \| |
| PSAT8 | (CL424)   \| size \| 55 \| \| --- \| --- \| \| size_real \| 55 \| \| ecount \| 410 \| \| supercluster \| 346 \| \|  \|  \| \| pair_completeness \| 0.617647058823529 \| \| pbs_score \| 0.0 \| \| TR_score \| 0.7849662162162162 \| \| TR_monomer_length \| 297.0 \| \| loop_index \| 0.8 \| \| satellite_probability \| 0.0307462659240343 \| | (CL373)  The cluster was not identified as putative satellite in the TAREAN report of this run, but homologous sequences were identified in contigs of this cluster. | (CL374)   \| size \| 147 \| \| --- \| --- \| \| size_real \| 147 \| \| ecount \| 2135 \| \| supercluster \| 206 \| \| annotations_summary \|  \| \| pair_completeness \| 0.484848484848485 \| \| pbs_score \| 0.0 \| \| TR_score \| 0.6731873803379672 \| \| TR_monomer_length \| 294.0 \| \| loop_index \| 0.931972789115646 \| \| satellite_probability \| 0.00378771683663762 \| | (CL280)   \| size \| 199 \| \| --- \| --- \| \| size_real \| 199 \| \| ecount \| 5834 \| \| supercluster \| 161 \| \| annotations_summary \| 0.50% Class_I/LTR/Ty3_gypsy/chromovirus/CRM:Ty3-INT \| \| pair_completeness \| 0.542635658914729 \| \| pbs_score \| 0.0 \| \| TR_score \| 0.7002277574924984 \| \| TR_monomer_length \| 298.0 \| \| loop_index \| 0.819095477386935 \| \| satellite_probability \| 0.0152157294996193 \| |
