# Supplementary material for: The nature and organization of satellite DNAs in Petunia hybrida, related, and ancestral genomes
Source: Front Plant Sci. 2023 Oct 6;14:1232588. doi: 10.3389/fpls.2023.1232588 (PMC10587573; doi:10.3389/fpls.2023.1232588)
Supplement: Supplementary file 1 [file DataSheet_1.zip › Table S3.docx]

**Alisawi et al. Petunia satellite repeats**

**Supplementary material**

**Table S3.** Number of raw reads mapped to the consensus reference sequences of the seven satellite repeats *P. hybrida* (PhybR27), *P. axillaris (PaxiN), P,inflata (PinfS6)* and *P. parodii (PparS7* with 0%, 5%, 10% and 20% mismatch to the reference. Total reads used for the mapping are given at the bottom,

|  | **Assembled reads** | | | | | | | | | | | | | | | |
| --- | --- | --- | --- | --- | --- | --- | --- | --- | --- | --- | --- | --- | --- | --- | --- | --- |
|  | ***PhybR27*** | | | | ***PaxiN*** | | | | ***PinfS6*** | | | | ***PparS7*** | | | |
|  | **0%** | **5%** | **10%** | **20%** | **0%** | **5%** | **10%** | **20%** | **0%** | **5%** | **10%** | **20%** | **0%** | **5%** | **10%** | **20%** |
| **PSAT1** | 59,862 | 369,037 | 394,039 | 432,140 | 56,598 | 277,076 | 318,237 | 322,547 | 35,523 | 288,048 | 320,087 | 323,613 | 18,142 | 102,497 | 111,944 | 119,213 |
| **PSAT3** | 6,958 | 49,684 | 113,922 | 190,097 | 12,688 | 90,519 | 315,753 | 405,411 | 3,022 | 17,024 | 67,733 | 131,713 | 3,170 | 11,457 | 63,019 | 87,826 |
| **PSAT4** | 2,318 | 44,272 | 98,775 | 174,802 | 285 | 26,011 | 77,344 | 115,717 | 465 | 30,276 | 84,512 | 113,435 | 757 | 15,402 | 42,906 | 64,988 |
| **PSAT5** | 62,927 | 108,876 | 137,736 | 186,015 | 19,575 | 41,005 | 49,607 | 104,225 | 7,865 | 22,868 | 34,152 | 64,845 | 8,054 | 23,867 | 36,496 | 63,402 |
| **PSAT6** | 16,325 | 51,230 | 78,836 | 157,924 | 11,811 | 33,533 | 63,726 | 113,929 | 6,534 | 21,762 | 39,294 | 57,901 | 6,295 | 18,108 | 36,822 | 75,148 |
| **PSAT7** | 2,058 | 13,013 | 35,703 | 98,971 | 3,185 | 14,666 | 142,230 | 401,936 | 261 | 18,716 | 54,930 | 131,713 | 1,251 | 3,789 | 22,052 | 93,681 |
| **PSAT8** | 4,573 | 13,265 | 17,277 | 38,174 | 3,613 | 11,286 | 13,585 | 49,085 | 3,006 | 7,965 | 10,237 | 24,835 | 3,733 | 8,456 | 10,309 | 19,160 |
| **Total reads** | 194,952,282 | | | | 166,007,734 | | | | 118,062,900 | | | | 79,576,106 | | | |
