# Supplementary material for: The nature and organization of satellite DNAs in Petunia hybrida, related, and ancestral genomes
Source: Front Plant Sci. 2023 Oct 6;14:1232588. doi: 10.3389/fpls.2023.1232588 (PMC10587573; doi:10.3389/fpls.2023.1232588)
Supplement: Supplementary file 1 [file DataSheet_1.zip › Figure S1.PDF]

Alisawi et al. Petunia satellite repeats

Supplementary data Figure S1

(A) **PSAT3**

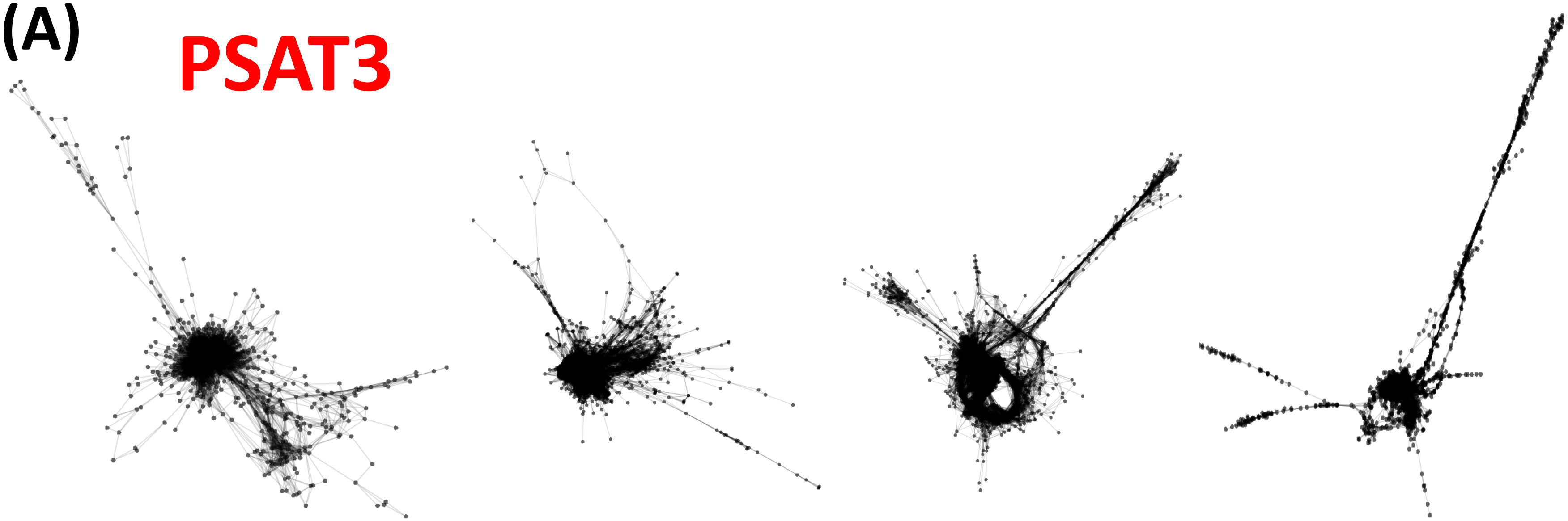

RE-*PhybR27*-CL101 RE-*PaxiN*-CL 58 RE-*PinfS6*-CL 103 RE-*Ppar*-CL117

Figure S1: Cluster graphs from the Repeat Explorer report of raw reads of Phyb27, PaxiN, PinfS6 and PparS7. More information about the clusters is available in Supplementary Data S1.

(A) PSAT3, (B) PSAT4, (C) PSAT5, (D) PSAT6, (E) PSAT7 and (F) PSAT8.

Note that *in PinfS6* the Cluster for PSAT7 is the same as for PSAT3.

Alisawi et al. Petunia satellite repeats

Supplementary data Figure S1 continued

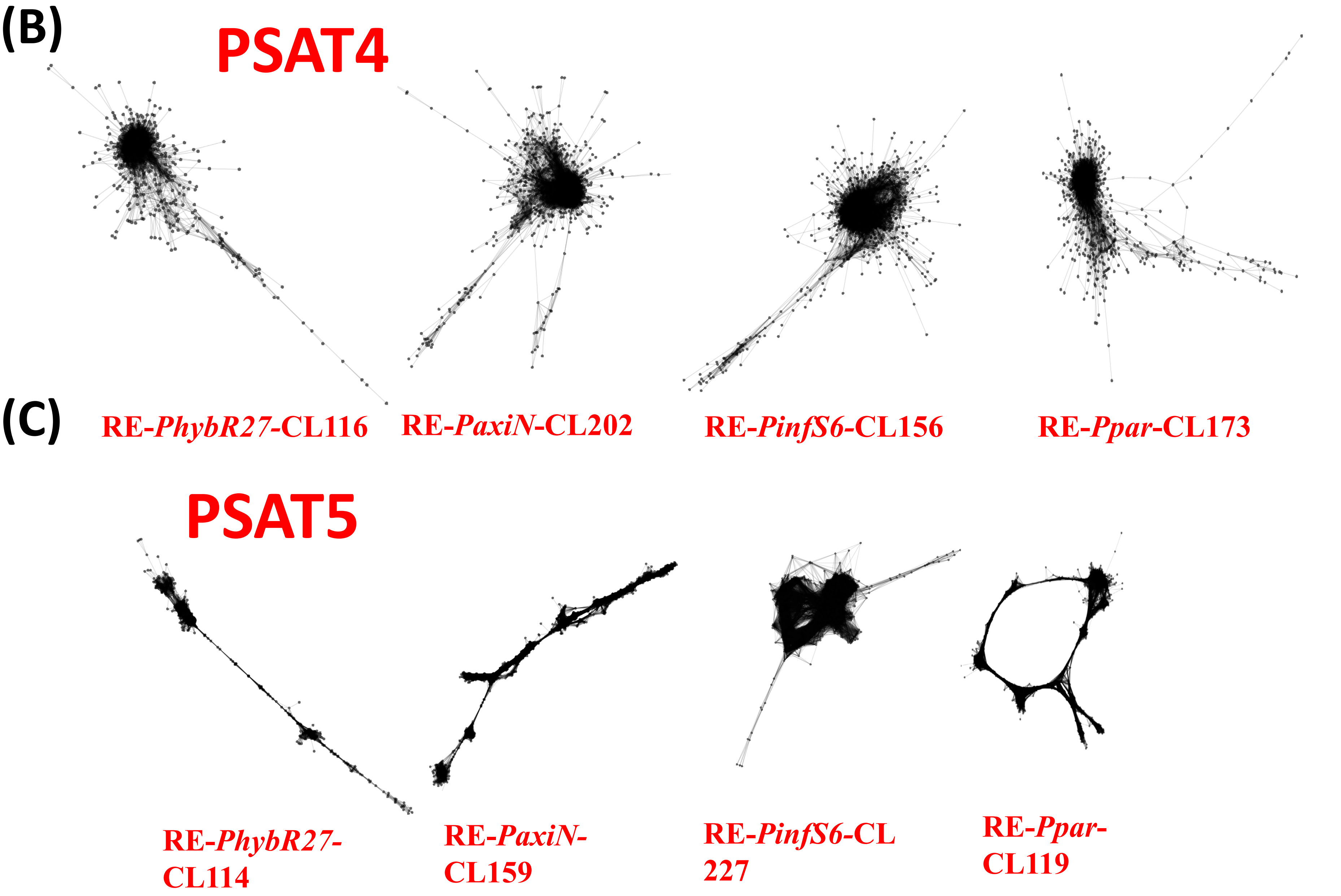

Supplementary data Figure S1 continued

(D)

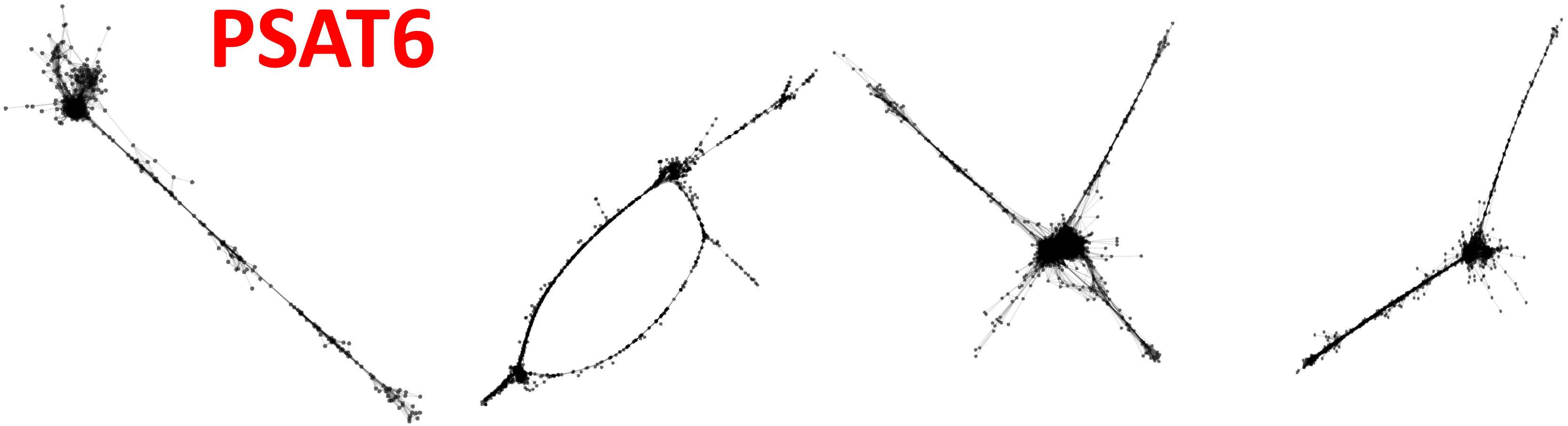

(E) RE-*PhybR27*-CL 145 RE-*PaxiN*-CL168 RE-*PinfS6*-CL222 RE-*Ppar*-CL175

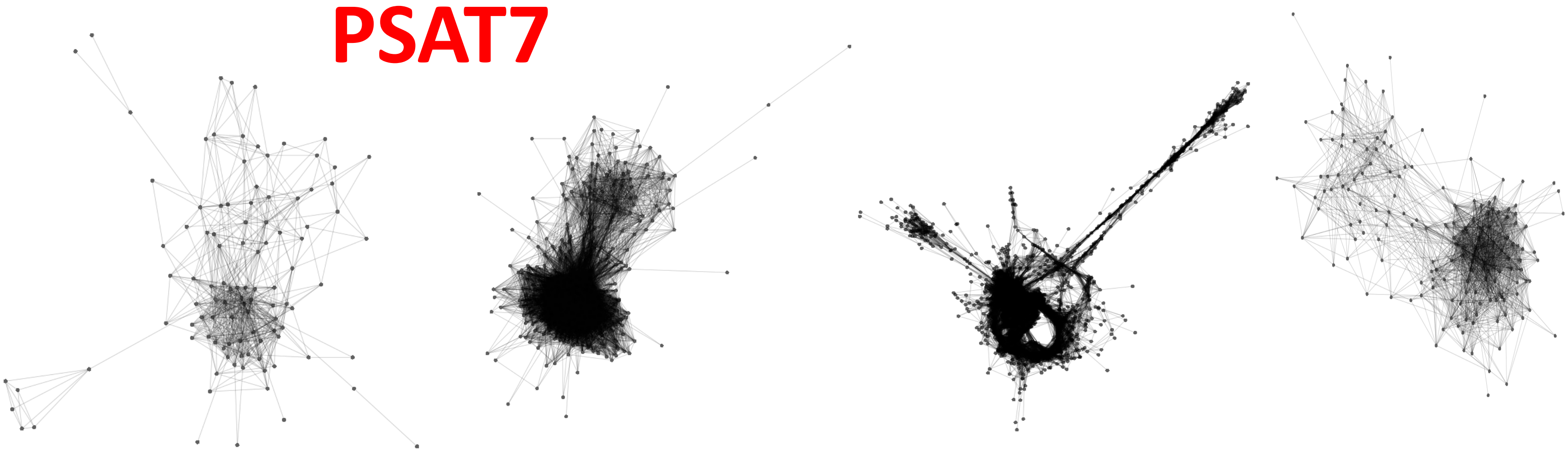

RE-*PhybR27*-CL302 RE-*PaxiN*-CL 290 RE-*PinfS6*-CL 103 RE-*Ppar*-CL299

Supplementary data Figure S1 continued

(F) PSAT8

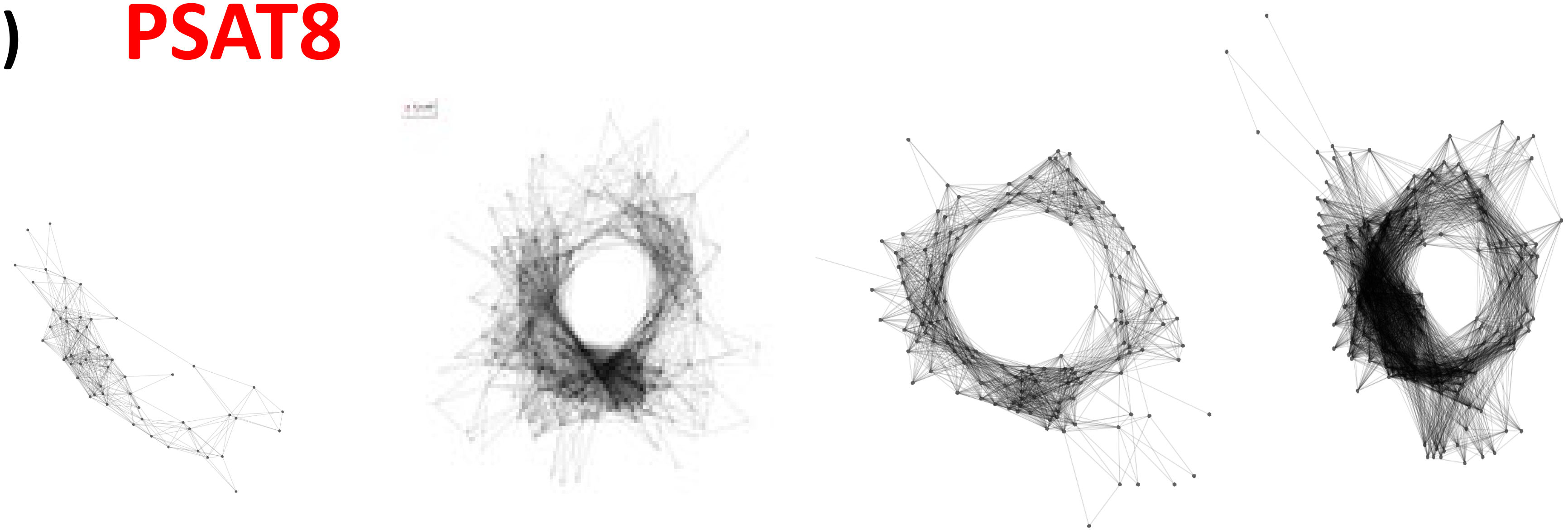

RE-*PhybR27*-CL424 | RE-*PaxiN*-CL373 | RE-*PinfS6*-CL 374 | RE-*Ppar*-CL280

Figure S1: Cluster graphs from the Repeat Explorer report of raw reads of Phyb27, PaxiN, PinfS6 and PparS7. More information about the clusters is available in Supplementary Data S1.

(A) PSAT3, (B) PSAT4, (C) PSAT5, (D) PSAT6, (E) PSAT7 and (F) PSAT8.

Note that *in PinfS6* the Cluster for PSAT7 is the same as for PSAT3.
