# Supplementary material for: The nature and organization of satellite DNAs in Petunia hybrida, related, and ancestral genomes
Source: Front Plant Sci. 2023 Oct 6;14:1232588. doi: 10.3389/fpls.2023.1232588 (PMC10587573; doi:10.3389/fpls.2023.1232588)
Supplement: Supplementary file 1 [file DataSheet_1.zip › Figure S2.pptx]

## Slide 1
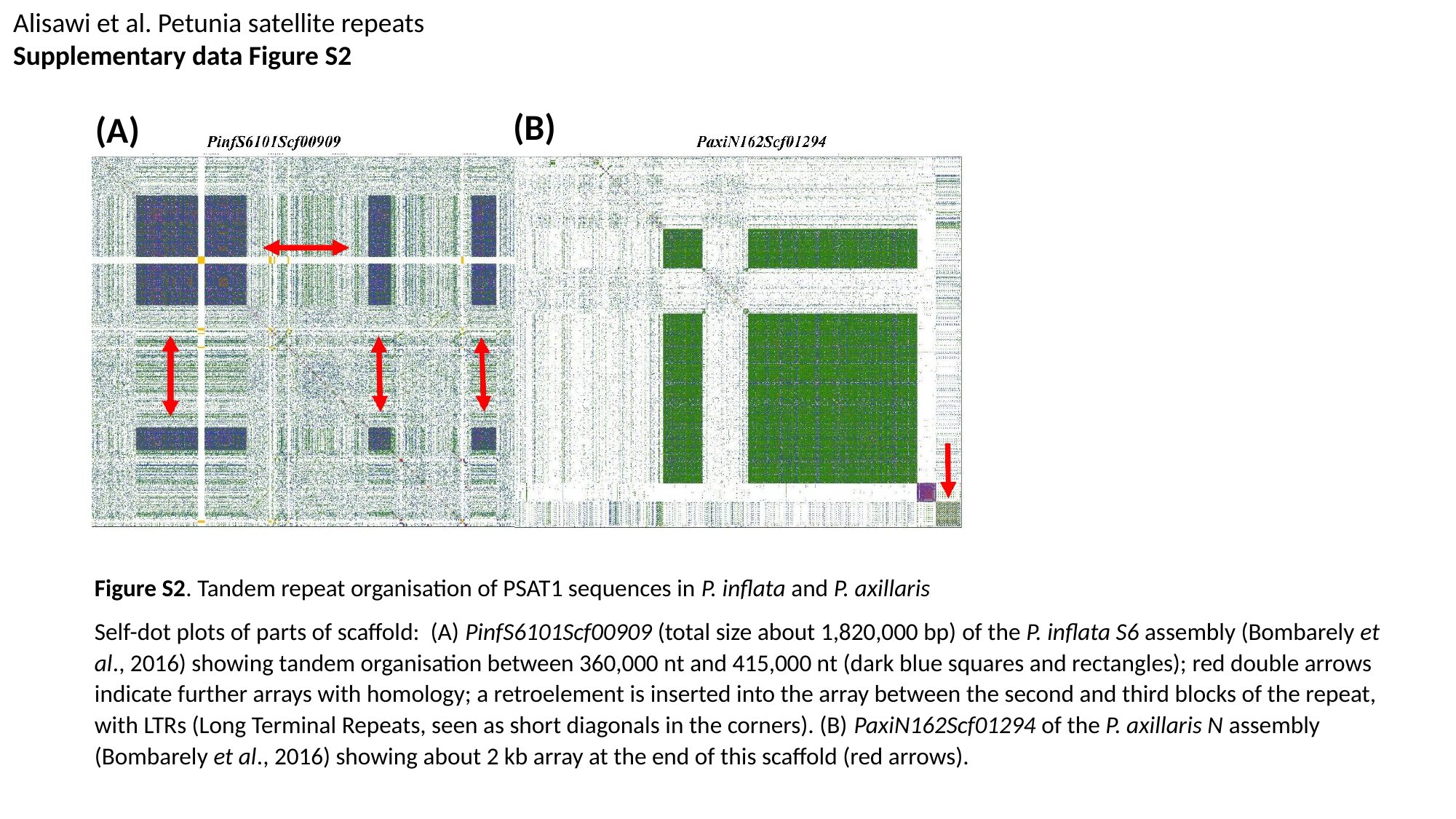

Alisawi et al. Petunia satellite repeats
Supplementary data Figure S2
(B)
(A)
Figure S2. Tandem repeat organisation of PSAT1 sequences in P. inflata and P. axillaris
Self-dot plots of parts of scaffold: (A) PinfS6101Scf00909 (total size about 1,820,000 bp) of the P. inflata S6 assembly (Bombarely et al., 2016) showing tandem organisation between 360,000 nt and 415,000 nt (dark blue squares and rectangles); red double arrows indicate further arrays with homology; a retroelement is inserted into the array between the second and third blocks of the repeat, with LTRs (Long Terminal Repeats, seen as short diagonals in the corners). (B) PaxiN162Scf01294 of the P. axillaris N assembly (Bombarely et al., 2016) showing about 2 kb array at the end of this scaffold (red arrows).

## Slide 2
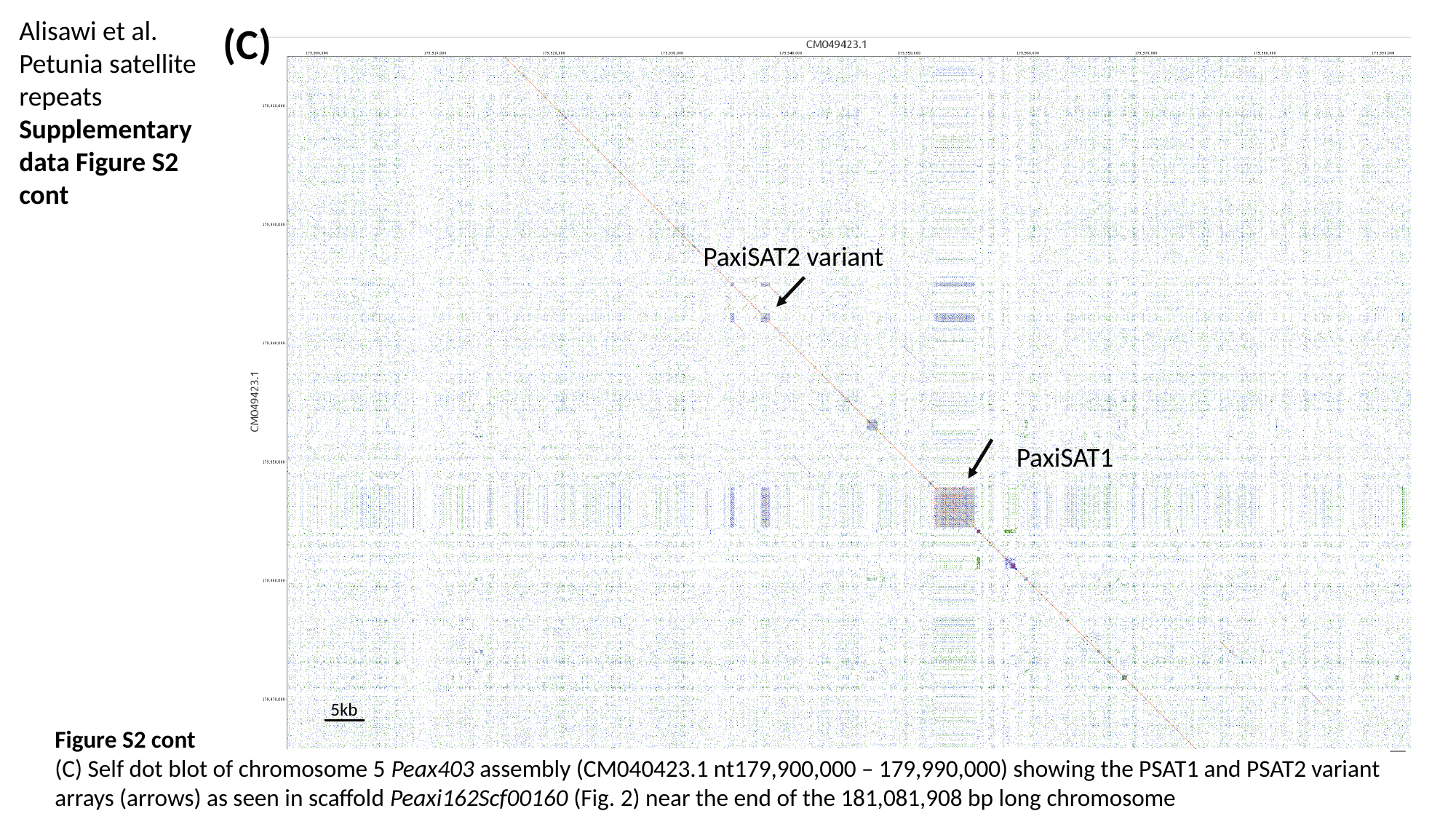

Alisawi et al. Petunia satellite repeats
Supplementary data Figure S2 cont
(C)
PaxiSAT2 variant
PaxiSAT1
5kb
Figure S2 cont
(C) Self dot blot of chromosome 5 Peax403 assembly (CM040423.1 nt179,900,000 – 179,990,000) showing the PSAT1 and PSAT2 variant arrays (arrows) as seen in scaffold Peaxi162Scf00160 (Fig. 2) near the end of the 181,081,908 bp long chromosome
