# Supplementary material for: The nature and organization of satellite DNAs in Petunia hybrida, related, and ancestral genomes
Source: Front Plant Sci. 2023 Oct 6;14:1232588. doi: 10.3389/fpls.2023.1232588 (PMC10587573; doi:10.3389/fpls.2023.1232588)
Supplement: Supplementary file 1 [file DataSheet_1.zip › Table S6.PDF]

Alisawi et al. Petunia satellite repeats **Supplementary material**

Table S6: Repeat sequences identified as putative satellites in the TAREAN and Repeat Explorer Reports of *P. inflata* S6 raw reads (*PinfS6*).

PinfSATs, consensus sequences, monomer lengths, selected extracted contigs. Repeats that were not found in the TAREAN report as putative satellites, had no consensus sequence; contigs of clusters were searched to find them.

Repeat	Cluster, TAREAN consensus and extract contig sequence<br>.	Comment	FISH
**PinfSAT1**<br><br>168bp monomer	Cluster CL63 (168bp)<br>TAREAN consensus, high confidence putative satellite, 0.27% of genome<br>CTTCAGAAAGCACGAACTACCAGATTTTTCAAAAAACTGAGTACTAGCCCATTTTTCTTTACAAC<br>GCCTACCTCTTTTTCATATGTTCGCTA**TAG**AAAG**T**GCAAACTA**T**CCCTTTTTATAGCATTTCTTG<br>ACAAAATTTTGAATTTTCATTTTTTGAACAGTCCGCTG<br>CL63 Contig11 extraction (504bp) with three 168bp monomers<br>**CTTCAGAAAGCACGAACTACCAGATTTTTCAAAAAACTGAGTACTAGCCCATTTTTCTTTACAAC**<br>**GCCTACCTCTTTTTCATATGTTCGCTATAGAAAGTGCAAACTATCCCTTTTTATAGCATTTCTTG**<br>**ACAAAATTTTGAATTTTCATTTTTTGAACAGTCCGCTG**<br>CTTCAGAAAGCACGAACTACCAGATTTTTCAAAAAACTGAGTACTAGCCCATTTTTCTTTACAAC<br>GCCTACCTCTTTTTCATATGTTCGCTATAGAAAGTGCAAACTATCCCTTTTTATAGCATTTCTTG<br>ACAAAATTTTGAATTTTCATTTTTTGAACAGTCCGCTG<br>**CTTCAGAAAGCACGAACTACCAGATTTTTCAAAAAACTGAGTACTAGCCCATTTTTCTTTACAAC**<br>**GCCTACCTCTTTTTCATATGTTCGCTATAGAAAGTGCAAACTATCCCTTTTTATAGCATTTCTTG**<br>**ACAAAATTTTGAATTTTCATTTTTTGAACAGTCCGCTG**	Primers that were used to clone the repeat from *P. axillaris* Forward (Scf160-72F: CCGAAAGCGCAAACTA TCCC) underlined. Reverse Primer (Scf160-26R: AAAAAGAGGTAGGCG TTGAAG) double underlined In cyan the part missing from the PaxiSAT1 clone	Signal on the ends of all chromosomes except the short arm of Chrs II and III

**PinfSAT3**<br>51bp<br>monomer	**Cluster 103**<br>TAREAN consensus (51bp), low confidence putative satellite, 0.17% of genome<br>TTGTCACTCTAACAAGTATAGTTGGTCATTTCTAGTGATAATGATCATCAT<br>CL103 Contig 91 (514bp, with ten 51bp monomers)<br>..TGAGTGGAAATGGCCAATTACACTTGTTATAGTGAGAAATGATGATCAT<br>TATGACTAGAAATGACCCACTATACTTGTAAGAGTGACAAATGATGATCAT<br>TATGAGTAGAAATGGCCAATTACACTTGTTAGAGTGACAAATGATGATGAT<br>TATCACTATAAATGACCAATTATACTTGTTAGAGTGACAAATGATGATCAT<br>TATGAGTAGAAATGGCCAATTACACTTGTTATAGTGAGAAATGATGATCAT<br>TATGACTAGAAATGACCCACTATACTTGTAAGAGTGACAAATGATGATCAT<br>TATCACTAGAAATGACCAATTATACTTGTTAGAGTGACAAATGATTATCGT<br>TATCACTAGAAAGGACCCATTATACTTGTAAGAGTGACAAGTGATGATCAT<br>TTTCACTAGAAATGACCAATTATACTTGTTAGAGTGACAAATGATGATCAT<br>TATGAGTAGAAATGGCTAATTACCCTGGTTACAGTGACAAATAATGATGAT<br>TATCAC<br>OligoFISH probe (51bp)<br>TCACTAGAAATGACCAATTATACTTGTTAGAGTGACAAATGATGATCAT<br>TA	Cluster CL103 contains PinfSAT3 and PinfSAT7 sequences in different contigs and the combined TAREAN consensus sequence hence fits badly to PinfSAT3.<br>OligoFISH probe -= monomer	FISH: FigS3J<br>Strong signal on a pair of unequal armed chromosome (II or III)

**PinfSAT4**<br><br>113bp<br>monomer	**Cluster CL156**<br>TAREAN consensus (113bp): Low confidence putative satellite, 0.12% of genome<br>ACC**C**TTTTGGTATACTGTATACTCTTTTGGTATACCCTTTTGTGTTTGGAT**CA**AAGTGTGCG<br>AT.GCTACCGAGCGAATAAATATTTCAGTTTTTGTTGTTTTGAACAAAGTAT<br>CL156 Contig17 extraction(225bp) with 114bp matching the TAREAN consensus (yellow)<br>AAATGAGTATTTCAATTATTGTTATTTTGAACAAAATAT<br>ACC**T**TTTTGGTATACTGTATACTCTTTCGGTATACCTTGTTATGTTTGGAT**AA**AAGTGTGAGATA<br>CTATCGAGCAAATAAAATATTTCAGTTTTTGTGTTTGTGAACAAAGTAT<br>GCCTTTTTGGAGAAAGAAAAAAAATGAGTATAATAAGATTACGGAGAAAGAAAATAAATAAAAAA<br>ATAAAAA<br>OligoFISH probe (55bp)<br>CC**T**TTTTGGTATACTGTATACTCTTTCGGTATACCTTGTTATGTTTGGAT**CG**AAG	TAREAN consensus in yellow<br>Oligo FISH probe underlined<br>Mismatches in bold	Dispersed and weak signal on all chromosomes (not shown)
**PinfSAT5**<br><br>**100bp**<br>**monomer**	**Cluster CL 227**<br>**TAREAN consensus (22bp): Low confidence putative satellite, 0.057% of the genome**<br>**TCGGCTTAGTCGCTCGGCCGCT (or CGGCTTAGTCGCTCGGCCGCTT)**<br>**CL227 Contig 23 extraction (400bp)**<br>**AATCAAATATTTATGTATGTT**<br>**ATGCATCAAAAAGCTT**<br>**TGGCTTAGTCGCTCGGCCCCTT**<br>**TGGCTTAGTCCCTCGGCCGCTTCGGCTCAAATATTTCCTACAATCAAATATTTATGTATGTT**<br>**ATGCATCAAAAAGCTT**<br>**TGGCTTAGTCGCTCGGCCCCTT**<br>**TGGCTTAGTCCCTCGGCCGCTTCGGCTCAAATATTTCCTACAATCAAATATTTATGTATGTTATG**<br>**CATCAAAAAGCTT**<br>**TGGCTTAGTCGCTCGGCCCCTT**<br>**TGGCTTAGTCCCTCGGCCGCTTCGGCTCAAATATTTCCTACAATCAAATATTTATGTATGTT**<br>**ATGCATCAAAAAGCTT**<br>**TGGCTTAGTCGCTCGGCCCCTT**<br>**TGGCTTAGTCCCTCGGCCGCTTCGGCTCAAATATTTCCTAC**<br>**oligoFISH probe revers (49bp)**<br>**CGGCCGCTTCGGCTCAAATATTTTCTACAATCAAATATTTATGTATGTT**	Four repeat units of 100bp (yellow and grey) are found that have the 78bp monomer from PhybPSAT5, PaxiSAT5 and PparSAT5, plus a 22bp internal duplication (highlighted cyan) that forms the basis of the TAREAN consensus<br>OligoFISH probe underlined	Fish Fig 2I.<br>Strong signal on 1 pair at the end, probably II or III

**PaxiSat6**<br><br>78 bp monomer with 39bp A and A' subrepeat	**Cluster CL222**<br>TAREAN consensus (39bp): Low confident putative satellite, 0.06% of genome<br>TCGAAAAGGAAATGATCGCTATCTTTTAGCGAAACTGAC<br>**Reverse**<br>..............................GTCAGTTTC<br>GCTAAAAGATAGCGATCATTTCCTTTTCGA<br>CL222 contig 16 extraction (304bp reverse) with seven 38bp monomers<br>.....................CCTTTTTGAGTCAGTTTC<br>GCTAAAAGATAGCG**AT**CATTTCCTTT**TCGA**GTCAGTTTC<br>GCTCAAAGATAGCG.**T**CATTTCCTTT**CCAT**GTCAAGATC**T**<br>GCTAAAAGATAGCG**AT**CATTTCCTTT**TCGA**GTCAGTTTC<br>GCTCAAAGATAGCG.**T**CATTTCCTTT**CCAT**GTCAAGATC**T**<br>GCTAAAAGATAGCG**AT**CATTTCCTTT**CCGA**GTCAATTTC**T**<br>GCTAAAAGATAGCA**AC**CATTTCCCTT**TTGA**GTCAGTTTC<br>GCTAAAAGATAGCA**CC**CATTTCCTTT**CCGA**GTCAATTTCT<br>GCTAAAAGATA<br>Oligo FISH probe (38bp) reverse<br>GCTAAAAGATAGCGATCATTTCCTTTTCGAGTCAGTTT	Monomer unit of 78bp made up of A(39bp)+A'(39bp) with some variation<br>OligoFISH probe underlined in first instance only	FISH, Fig S3R<br>2 strong and 4 weaker near the centromere of equal armed chromosomes
**PSAT7**<br>51bp monomer	**Cluster 103**<br>Tarean consensus (51bp)<br>TTGTCACTCTAACAAGTATAGTTGGTCATTTCTAGTGATAATGATCATCAT<br><br>CL103 contig 83 (156bp) with three 51bp monomers<br>GTGATGATCACCAATA<br>TTTATCACTATAACTAGTATAATTGGTCATGTGTAGTGATAATCATCATCA<br>TTTATCACTCTTACTTGTATATTTGGTCATGTCTAGTGATGATCATCATCA<br>TTTATCACTCTTACTTGTATAATTGGTCATGTATAGTG<br>Oligo FISH probe (51bp) reverse<br>TTTATCACTCTTACTTGTATATTTGGTCATGTCTAGTGATGATCATCATCA	Cluster 103 contains Pinf3SAT3 and PInfSAT7, but in different contigs. TAREAN consensus is hence not a good fit for either<br>OligoFISH probe is the monomer	FISH<br>Strong signal as with PSAT3 plus a few minor sites

**PinfSAT8**<br>294bp<br>monomer	Cluster CL374<br>Tarean consensus (294bp)<br>CCCCCCTTTCTTTAAAGCAAGTTCGTCCCCGAACTTCGCATGGTGTAGCCAGGAATCGAACCCGG<br>GTTGGCTCTGATACCAAACTTTAAGAGTGCACTGCAACCATTTCTACCAAAAGCCATAGCTGATG<br>GTAGAGGCGCAACGCAACTCTTATACCGCCGGATCAACCCCAAATCGAGGTCCGTCCCTGACACC<br>ACCATGACCACCCCGAGCAACGCAACTCCTCCAGGGGGTGGACCCCACAACGGGCGGCTCCTTAT<br>GATGTGGGCCCAATCCTTGATAGCCCTGCATCAT<br>Tarean consensus (294bp) reverse<br>ATGATGCA<br>GGGCTATCAAGGATTGGGCCCACATCATAAGGAGCCGCCCGTTGTGGGGTCCACCCCCT<br>GGAGGAGTTGCGTTGCTCGGGGTGGTCATGGTGGTGTCAGGGACGGACCTCGATTTGGG<br>GTTGATCCGGCGGTATAAGAGTTGCGTTGCGCCTCTACCATCAGCTATGGCTTTTGGTA<br>GAAATGGTTGCAGTGCACTCTTAAAGTTTGGTATCAGAGCCAACCCGGGTTCGATTCCT<br>GGCTACACCATGCGAAGTTCGGGGACGAACTTGCTTTAAAGAAAGGGGGG<br>CL374 contig 7 extraction (401bp)<br>GCTACACCATGCGAAGTTCGGGGACGAACTTGCTTTAAAGAAAGGGGGGATGATGCA<br>GGGCTATCAAGGATTGGGCCCG<br>GGGCTATCAAGGATTGGGCCCACATCATAAGGAGCCGCCCGTTGTGGGGTCCACCCCCT<br>GGAGGAGTTGCGTTGCTCGGGGTGGTCATGGTGGTGTCAGGGACGGACCTCGATTTGGG<br>GTTGATCCGGCGGTATAAGAGTTGCGTTGCGCCTCTACCATCAGCTATGGCTTTTGGTA<br>GAAATGGTTGCAGTGCACTCTTAAAGTTTGGTATCAGAGCCAACCCGGGTTCGATTCCT<br>GGCTACACCATGCGAAGTTCGGGGACGAACTTGCTTTAAAGAAAGGGGGGATGA<br>TGCAGGGCTATCAAGGATTGGGTCCAAACTCA<br>Oligo FISH probe (54bp)<br>GGCTACACCATGCGAAGTTCGGGGACGAACTTGCTTTAAAGAAAGGGGGGATGA	Tarean consensus (yellow),there is a 22bp duplication (cyan) in the extracted Contig, oligo FISH probe underlined	No FISH signal
