# Supplementary material for: The nature and organization of satellite DNAs in Petunia hybrida, related, and ancestral genomes
Source: Front Plant Sci. 2023 Oct 6;14:1232588. doi: 10.3389/fpls.2023.1232588 (PMC10587573; doi:10.3389/fpls.2023.1232588)
Supplement: Supplementary file 1 [file DataSheet_1.zip › Table S7.PDF]

Supplementary material

Table S7: Repeat sequences identified as putative satellites in the TAREAN and RepeatExplorer Report of *P. parodii* S7 raw reads (*PparS7*). PparSATs, consensus sequences, monomer lengths, selected extracted contigs. Repeats that were not found in the TAREAN report as putative satellites, had no consensus sequence; contigs of clusters were searched to find them.

Repeat	Cluster, TAREAN consensus and extract contig sequence.	Comments	FISH
**PparSAT1**<br>168bp<br>Monomer	**Cluster CL147**<br>TAREAN consensus High confidence putative satellite;0.15% of genome<br>GTTCAAAAAATGAAAATTCAAAATTTTGTCAAGAAATGCTATAAAAAGGTATAGTTT<br>GCACTTTCTATAGCGAACATATGAAAAAGAGGTAGGCGTTGTAAAGAAAAATGGGCT<br>AGTACTCAGTTTTTTGAAAAATCTGGTATTTCGTGCATTCTGAAGGTGCGGACT<br><br>CL147 Contig 19 Contig extraction (504bp, with three 168bp monomer<br>AAAAAATGAAAATTCAAAATTTTGTCAAGAAATGCTATAAAAAGG**T**ATAGTTT<br>GCACTTTCTATAGCGAACATATGAAAAAGAGGTAGGCGTTGTAAAGAAAAATGGGCT<br>AGTACTCAGTTTTTTGAAAAATCTGGTATTTCGTGCATTCTGAAGGTGCGGACT<br><br>GTTCAAAAAATGAAAATTCAAAATTTTGTCAAGAAATGCTATAAAAAGG**T**ATAGTTT<br>GCACTTTCTATAGCGAACATATGAAAAAGAGGTAGGCGTTGTAAAGAAAAATGGGCT<br>AGTACTCAGTTTTTTGAAAAATCTGGTATTTCGTGCATTCTGAAGGTGCGGACT<br><br>GTTCAAAAAATGAAAATTCAAAATTTTGTCAAGAAATGCTATAAAAAGG**T**ATAGTTT<br>GCACTTTCTATAGCGAACATATGAAAAAGAGGTAGGCGTTGTAAAGAAAAATGGGCT<br>AGTACTCAGTTTTTTGAAAAATCTGGTATTTCGTGCATTCTGAAGGTGCGGACT<br><br>GTTC	Forward Primer (Scf160-72F: CCGAAAGCGCAAACT ATCCC) underlined; Rev compl. GGGATAGTTTGCGCT TTCGG<br>Reverse Primer (Scf160-26R: AAAAAGAGGTAGGC GTTGAAG) Double underlined<br>Mismatches in bold<br>Cyan indicates the part missing from the PSAT1 clone	Signal on all ends, except chr II and II short arm

 PparSAT3<br><br>51bp<br>monomer	Cluster CL117<br>No TAREAN consensus<br>CL117 Contig15 (562bp) with eight 51bp monomers<br>TGAGAAATGATGATCCATACATATAGTCTTGTTTCGAACATCAAATAGATGTTTG<br>TCACAATTTATGTGTCGTTCCGAACGCGGTGTTCAATTGTTTCTATCTTTATCAT<br>ACTTTTCACTGTTAGAGTAGTTAGAGTGACAAATTATGATGGTTATA<br><br>TCACTA**T**AA**G**TGA**GG**AATTATACTTGTAAGAGTGA**TAAAT**T**C**AT**CTA**C**G**TTA**A**<br>TCACAA**A**A...GACCA**G**TTA**G**A**A**T**A**GT**T**AGA**C**TGA**G**AA**T**TGATGATCATT**G**<br>TCACTAGAAATGGCCAATTATAC**C**TGT**A**AGAGTGACAA**G**TGATGATCATTA<br>TCACTAGAAATGACCAATTATACTTGTTAGAGTGACAAATGATGATCATTA<br>TCACTAGAAATGACCAATTA**C**ACTTGTTAGAGTGACAAATGA**C**GATCATTA<br>T**G**ACTAGAAATGACC**C**ACTAT**C**CTTGT**A**AGAGTGACAAATAATGATCATTA<br>TCACTA**T**AAATG**G**CCAATTATACTTGTTATAGTGA**G**AAATGATGATCATTA<br>TGAGT**G**GAAAT**TG**CCAATTA**C**ACT**G**GG**G**ATA**T**T**A**ACAAATGATGATCAT<br>Oligo FISH probe (51bp)<br>TCACTAGAAATGACCAATTATACTTGTTAGAGTGACAAATGATGATCATTA	The cluster did not appear in the report but was found and extracted from contigs of CL117<br><br>Imperfect and near perfect monomers (yellow and grey alternating)<br>OligoFISH probe = monomer	FISH: 4 strong and 2 weak signals near centromeres

**PparSAT4**<br><br>113bp monomer:	Cluster CL173<br>TAREAN consensus (113bp) Low confidence putative satellite, 0.091% of the genome<br>ACTGAAATATTTATTCGCTCGGTAGCATCGCACACTTGGATCCAAACACAAAAGG<br>GTATACCAGAAGAGTATACAGTATACCAAAAGGGTATACTTTGTTCAAAACAACAAAs<br>A<br>CL173 Contig11 extraction (578bp) including four 113bp monomers<br>CCAAAAGGGTATACTTTGTTCAAAACAACAAAACATTTTCTTATTTTTTTATTTATTT<br>TCTTTCTCCGTAAACTTATTATACTCTTTTTTTTTCTTTCTCCAAAAAGGCATACTT<br>TGTTCACAAACACAAAA<br>ACTGAAATATTTTATTTGCTCGATAGTATCTCACACTT**TT**ATCCAAACATAA**A**A**C**GG<br>TATACC**A**AAAGAGTATA**TGA**TATAC**A**AAAAAGGTATACTTCTTTAAAAATAATAAAA<br>ATTAAAATATTTATGTGCTCTGAAACATCTCGCACTTCGATCC**G**AACATAACAAGG<br>TATACCGAAAGAGTATACAGTATACCAAAAAGGTATATTTTGTTCAAAATAACAATA<br>ATTGAAATACTCATTTGCTCGATAGCATCGCACACTT**T**GATCCAAACA**C**AA**A**A**G**GG<br>TATACC**A**AAAGAG**A**ATACA**A**TATACCAAAAAGGTATACTTCTTTAAAAATAATAAAA<br>ATTAAAATATATATGTCTTCTGAAACATTTTGCACTTCGAT**T**C**G**AACA**C**AA**A**A**G**GG<br>TATACC**A**GAAGAGTATA**A**AGTATACCAAAAGGGTATA<br>CCAAAAGGGTATACTTTGTTCAAAATAATAAAAACTGAAATATTT<br>OligoFISH probe (55bp) Reverse<br>CTTCGATCCAAACATAACAAGG<br>TATACCGAAAGAGTATACAGTATACCAAAAAGG	Alternating 113bp monomers in yellow and grey<br>oligoFISH probe underlined	Weak and dispersed FISH signal

**PparSAT5**<br><br>Monomer 78bp	Cluster CL119<br>No TAREAN consensus<br>CL119 Contig7 extraction (2353bp)<br>ATACCAAAAAAATATTATAATTTATTATAGAAAGACACAAATGATTGTAGCATGATTG<br>TATTATGTAAATTTAGAAAATAGATTAATATTTAAATAATAAAAAAGTAATAAAAAGT<br>ATTTAAAATACATAAATAATAATATAAATATTAAATAAATATAATAAAATACCAAAAA<br>TCACAAAAAAGTATTATAATTTATTATAGAAAGCCACAAATGATTGTAGCATGATTGT<br>ATTATGTAAATTTAGAAAATAAATAAATATTTAAATAATAAAAAAGTAATAAATAAAT<br>AAAAATATTATTTTGGTGGAGAAAAATATCAAGGGCATCGGGTAAGTACACGATACAC<br>GAAATGAGTATGAAAAGACATAAATGAGTAGACACCAATAGTCAAAAGAGATGCAATG<br>TTTTAGTTTGGCGGTGTTTCAGTATAAGAAACGTTTGCACCACATTATATATCGGTAC<br>GTACTCAATCGTGCACAACGGTACATATCGAACTTCTTTTTGGAACATAGAGAGACCG<br>TGCACTCGGCCACTACGGCTTAGTCCCT<br>CGGCCGCTTCGGCTCAAATATTTTCTACAATCAAATATTTATGTATGTTATGCATCAA<br>AAAGCTTCGGCTTAGTCGCT<br>CGGCCGCTTCGGCTCAACTATTTCCTACAATCAAATATTTATGTATGTTATGCATCAA<br>AACATCACGAAGCACCGGCT<br>TAGTAGTCCGGCCGCTTTGGCTTAGTACCTCGGCCGCTTCCGCTTACTTTGGCTTAGT<br>ATCTCGGCCGCTTCCGCCTACTTCGGCTTAGTAGTTCGGCCGCTTTGGCTTAGTCCCT<br>CGGCCGCTTCCGCTTACTTTGGCTTAGTATCTCGGCCGCTTCCGCCTACTTCGGCTTA<br>GCCGTTCGGCCGCTTCCGCCTACTTTGGCATAGTCTCTCGACCGCTTCGGCTTAGCCG<br>TTCGGCCGCTTCCGCCTACTTTGGCATAGTCTCTCGACCGCTTCGGCTTAGCCGTTCG<br>GCCGCTTTGGCTTAGTCCCTCGGCCGCTTCGGCTTACTTTGCCTTAGTCGCTCGGCCG<br>CTTCGGCTTACTTTGCCTTAGTCGCTCGGCCGCTTCGGCTTACTTTGCCTTAGTCGCT<br>CGGCCGCTTTGCCTTAGTCTCTCGGCCGCTTCGGCTTAGTAGTTCGGCCGCTTTGGCT<br>TAGTACCTCGGCCGCTTCCGCTTAGTCCCTCGGCCGCTTCCGCCTACTTTGGCTTAGT<br>CTCTCGACCGCTTCGGCTTAGCCGTTCGGCCGCTTTGGCTTAGTCCCTCAGCCGCTTC<br>GGCTTACTTTGCCTTAGTCGCTCGGCCGCTTTGGCTTACTTTGCCTTAGTCTCTCGGC<br>CACTTTGACTTAGTCTCT	This large contig of CL119 has several sub-repeats including 2 copies of the 78bp units (yellow and grey) found in the other 3 species and another similar, but longer 120bp repeat (olive and dark grey)<br><br>OligoFISh probe underlined	FISH:<br>2 signals at the end of Chr II or III. Some weak signal at several centromeres

	CGGCCGCTTCGGCTCAGCCGTTCGGCCGCTTCGGCTTAGTCCCTCGGCCGCTTCCGCC<br>TACTTTGGCATAGTCTCTCGACCGCTTCGGCTTAGCCGTTCGGCCGCTTTGGCTTAGT<br>CTCT<br>CGGCCGCTTCGGCTCAGCCGTTCGGCCGCTTCGGCTTAGTCCCTCGGCCGCTTCCGCC<br>TACTTTGGCATAGTCTCTCGACCGCTTCGGCTTAGCCGTTCGGCCGCTTTGGCTTACT<br>TTGC<br>CTTAGTCGCTCGGCCGCTTTGGCTTAGTCCCTCGGCCGCTTCGGCTTAGTTTGCCTTA<br>GTCGCTCGGCCGCTTTGCCTTAGTCTCTCGGCCGCTTCCGCCTAGCCGTTCGGCCGCT<br>TCGGCTTAGTCCCTCGGCCGCTTCCGCTTACTTTGGCTTAGTTGCTCGGCCGCTTTAG<br>CCTAGTCTCTCGGCCGCTTCCGCTTAGCCGTTCGGCCGCTTTGGCTTAGTCCCGGCTT<br>ACTTTGACTTAGTTGCTCGGCCGCTTTGGCTTAGTCTCTCGGCCGCTTCGGCTAAGCC<br>GTTCGGCCGCTTTGGCTTTGTCCCTCGGCCGCTTCCGCTTACTTTGGCTTAGCCGATC<br>GGCCGCTTCGGCTTAGCCGTTCGGCCGCTTTGGCTCAAATATTTCCTACAATCAAATA<br>TTTATGTATGTTATGCATAGAGACATCACGAAGCATCGGCTTAGCCTTTCGGCCGCTT<br>TGGCTTAGTCCCTCGGCCGCCTCCGCTTACTTTGACTTAGTTGCTCGGCCGCTTTGGC<br>TTAGTCTCTCGGCCGCTTTGGCTTAGGCAATCGGCCGCTTTGGCTTAGGCAATCGGCC<br>GCTTTGGCTTAGGCAATCGGCCGCTTGGGTCGCTTGGGCAAGTTTTTGTGTCATCATA<br>TTTCCCAACACTTAGTTTTTTTTTGTCCGCCAAGTGTAGGTTGAACCTCTTTACGAAA<br>ATATTAGAATAGTTGGATTGGAGGGGAGGGGGGGGGGGGGGACGAATCGGAGCGA<br><br>oligoFISH probe reverse (49bp)<br>CGGCCGCTTCGGCTCAAATATTTTCTACAATCAAATATTTATGTATGTT		

**PparSAT6**<br>78bp monomer with 39bp A and A' subunits	Cluster CL175:<br>No TAREAN consensus<br>CL175 Contig 13 (1737bp) including 44 units of subrepeats A (38bp) and A' (38bp)<br>ACCAACTTGAAAAGCGAGTGGTT<br>GCTATCTTTGAGC<br>ACAAAT GACTTGAACTGAATGTGAGTGCTATCTTCTAGC<br>AAAACT GACTTGAAAAGAAAATGATCGCTATCTTTTAGC<br>AGATCTTGACATGGAAAGGAAATGA CGCTATCTTTGAGC<br>GAAACT GACTCGAAAAGGAAATGATCGCTATCTTTGAGC<br>GAAACT GACTCGAAAAAGAAATGATCGCTATCTTTTAGC<br>AGATCTTGACATGGAAAGGAAATGA CGCTATCTTTGAGC<br>GAAACT GACTCGAAAAGGAAATGATCGCTATCTTTTAGC<br>AGATCTTGACATGGAAAGGAAATGA CGCTATCTTTGAGC<br>GAAACT GACTCGAAAAGGAAATGATCGCTATCTTTTAGC<br>AGATCTTGACATGGAAAGGAAATGA CGCTATCTTTGAGC<br>GAAACT GACTCGAAAAGGAAATGATCGCTATCTTTTAGC<br>AGATCTTGACATGGAAAAGAAATGA CGCTATCTTTGAGC<br>AAACT GACTCGAAAAGGAAATGATCGCTATCTTTTAGC<br>AGATCTTGACATGGAAAGGAAATGA CGCTATCTTTGAGC<br>GAAACT GACTCGAAAAGGAAATGATCGCTATCTTTTAGC<br>AGATCTTGACATGGAAAGGAAATGA CGCTATCTTTGAGCG<br>AAAACT GACTCGAAAAGGAAATGATCGCTATCTTTTAGC<br>AGATCTTGACATGGAAAGGAAATGA CGCTATCTTTGAGC<br>G AACT GACTCGAAAAGGAAATGATCGCTATCTTTTAGC<br>AGATCTTGACATGGAAAGGAAATGA CGCTATCTTTGAGC<br>GAAACT GACTCGAAAAGGAAAAAGATCGCTATCTTTTAGC<br>AGATCTTGACATGGAAAGGAAATGA CGCTATCTTTGAGC<br>GAAACT GACTCGAAAAGGAAATGATCGCTATCTTTTAGC<br>AGATCTTGACATGGAAAGGAAATGA CGCTATCTTTGAGC<br>GAAACT GACTCGAAAAGGAAATGATCGCTATCTTTTAGC<br>AGATCTTGACATGGAAAGGAAATGA CGCTATCTTTGAGC	Monomer of 76bp made up of A(39bp)+A'(39bp)<br>Oligo FISH probe underlined in A'	FISH<br>6 signals on Chr II and III and a more equal armed chr pair

	GAAACT GACTCGAAAAGGAAATGATCGCTATCTTTTAGC<br>AGATCTTGACATGGAAAGGAAATGA CGCTATCTTTGAGC<br>GAAACT GACTCGAAAAGGAAATGATCGCTATCTTTTAGC<br>AGATCTTGACATGGAAAGGAAATGA CGCTATCTTTGAGC<br>GAAACT GACTCGAAAAGGAAATGATCGCTATCTTTTAGC<br>AGATCTTGACATGGAAAGGAAATGA CGCTATCTTTGAGC<br>GAAACT GACTCGAAAAGGAAATGATCGCTATCTTTTAGC<br>AGATCTTGACATGGAAAGGAAATGA CGCTATCTTTGAGC<br>GAAACT GACTCGAAAAGGAAATGATCGCTATCTTTTAGC<br>AGATCTTGACATGGAAAGGAAATGA CGCTATCTTTGAGC<br>GAAACT GACTCGAAAAGGAAATGATCGCTATCTTTTAGC<br>AGATCTTGACATGGAAAGGAAATGA CGCTATCTTTGAGC<br>GAA CT GACTCGAAAAGGAAATGATCGCTATCTTTTAGC<br>AGATCTTGACATGAAAAAGAAATGA CGCTATCTTTGAGC<br>GAA CT GACTCGAAAAGGAAATGATCGCTATCTTTTAGC<br>AGATCTTGACATGAAAAAGAAATGA CGCTATCTTTGAGC<br>GAA CT GACTCGAAAAAAGAAATAATCGCTATCTTTGAGC<br>GAAACT GACTCGAAAAGGAAATGATG<br>OligoFISH probe (38bp)<br>AAACTGACTCGAAAAGGAAATGATCGCTATCTTTTAGC		

**PparSAT7**<br><br>51bp monomer	Cluster 299<br>TAREAN consensus (51bp) low confidence putative satellite; 0.014% of the genome<br>CTAACTTGTATATTTGGTCATGTCTAGTGATGATCATCATCATTTATCACT<br>CL299Contig1 extraction (1214bp) 23 monomers<br>ATAAGTAAGAGTGA<br>TAAATGATGATGATCATCTCTAAACGTGACCAAATATACAAGTGTGATAAA<br>TGAAGGTGATTATCACTACACATGACCAATTTTACTAGTTAGAGTGATAAA<br>TGATGGTGATAATCACTACACATGACCAATTATACTAGTTAGAGTGATAAA<br>CGATGGTGATTATCACTAGACACGACACATTGTACTAGTTAGAGTGATAAA<br>TTATGCGGATAATCACTAGACATGACCAAATCTACAAGTAAGAATGATAAA<br>TGATGGTGATCATCACTATACATGACCAATTATACTAGTTTAGAGTGATAAA<br>TGATG**G**TGATTATCACTAGACATGATCAATTATACTAGT**T**AG**T**G**G**GATAAC<br>TG**T**TG**G**TGATTAT**A**ACTA**C**A**A**ATGACCAATTATACTAGT**T**ATAGTGATAAA<br>TGATG**C**GGATAATCACTAGACATGACCAAATATACAA**C**T**T**AGATTGATAAA<br>TGAT**T**ATGATCATCACTAGACATGACCAAATATACAAG**G**TAGAGTGATAAA<br>TGATGATGATCGTCACTAGACATGACCAAATATACAAGTAAGAGTGATAAA<br>TGATGATGATCATCACTAGACATGACCAAATATACAAGTAAGAGTGATAAA<br>TGATGATGATCATCACTAGACATGACCAAATATACAAGTAAGAGTGATAAA<br>TGATGATGATCATCACTAGACATGACCAAATATACAAG**GT**AGAGTGATAAA<br>TGATGATGATCATCACTAGACATGACCAAATATACAAGTAAGAGTGATAAA<br>TGATGATGATCATCACTAGACATGACCAAATATACAAGTAACAGTGATAAA<br>T**A**ATGATGATCATCACTAGACACGACCAAATATACAA**AGT**AGAGTGA**C**AAA<br>TGATG TGATCATCACTAGAC**T**TGACCAAAT TACAAG**GT**AGAGTGATAAA<br>TGATGATGA**CA**ATCACTA**C**ACATGACCAAATATA**T**AAGTAAGAGTGATAAA<br>TGATGATGATCATCACTA**A**ACATGACCAAATATACAAGTAAG**T**GTGATAAA<br>TGAAGGTGAT**T**ATCACTA**C**ACATGACCAATTATAC**T**AGT**T**AGAGTGATAAA<br>**C**GATGGTGAT**T**ATCACTAGACA**C**GAC**AC**ATT**G**TAC**T**AGT**T**AGAGTGATAAA<br>TGATG**CG**GAT**A**ATCACTAGACATGA**TG**AATTACACTAGT**T**AG**T**GGGATAAC<br>TGTTGGTGATTATAACTACAAATGACCA<br>Oligo FISH probe (51bp)<br>TGATGATGATCATCACTAGACATGACCAAATATACAAGTAAGAGTGATAAA	51bp monomers (highlighted grey and yellow and the few mismatches in bold), variable (not highlighted and not all mismatches identified)<br>OligoFISH probe = monomer<br>High homology to PSAT3	FISH<br>One pair of signals near the centromere of an unequal armed chr. 2-4 weak signals mid arm of equal armed chr.

**PparSAT8**<br><br>298bp<br>monomer	**Cluster CL280**<br>TAREAN consensus (298bp), low confidence putative satellite, 0.017% of genome<br>ATCTTATACCGCCGGATCAACCCCAAATCGAGGTCCGTCCCTGACACCACCATGACCA<br>CCCCGAGCAACGCAACTCCTCCAGGGGGTGGACCCCACTACGGGCGGCTCCTTATGAT<br>GCGGGCCCAATCCTTGATAGCCCAGCTGCATCATCCCCCCTTTCTTTAAAGCAAGTTC<br>GTCCCCGAACTTCGCATGGTGTAGCCAGGAATCGAACCCGGGTTGGCTCTGATACCAA<br>ACTTTAAGAGAGGCACTGCAACCATTTCTACCAAAAGCCATAGCTGATGGTAGAGGCG<br>CAACTCAC<br>CL280 Contig1 extraction (469bp) with one and a half monomers., some mismatches and insertions<br>GGCCCAATCCTTGATAGCCCAGCTGCATCATCCCCCCTTTCTTTAAAGCAAGTTC<br>GTCCCCGAACTTCGCATGGTGTAGCCAGGAATCGAACCCGGGTTGGCTCTGATACCAA<br>ACTTTAAAGAGGGCACTGCAACCATTTCTACCAAAAGCCATAGCTGATGGTAGAGGCG<br>CAACGCAA<br><br>**C**TCTTATACCGCCGGATCAACCCCAAATCGAGGTCCGTCCCTGACACCACCATGACCA<br>CCCCGAGCAACGCAACTCCTCCAGGGGGTGGACCCCACTACGGGCGGCTCCTTATGAT<br>GCGGGCCCAATCCTTGATAGCCC<br>CGGGCCCAATCCTTGATAGCCC<br>AGCTGCATCATCCCCCCTTTCTTTAAAGCAAGTTC<br>GTCCCCGAACTTCGCATGGTGTAGCCAGGAATCGAACCCGGGTTGGCTCTGATACCAA<br>ACTTTAAGAGAGCACTGCAACCATTTCTACCAAAAG<br>Oligo FISH probe<br>GGCTACACCATGCGAAGTTCGGGGACGAACTTGCTTTAAAGAAAGGGGGGATGA<br>reverse<br>TCATCCCCCCTTTCTTTAAAGCAAGTTCGTCCCCGAACTTCGCATGGTGTAGCC	OligoFISH probe underlined.<br>Monomer in yellow and grey highlight	No clear FISH signal
