# Supplementary material for: The nature and organization of satellite DNAs in Petunia hybrida, related, and ancestral genomes
Source: Front Plant Sci. 2023 Oct 6;14:1232588. doi: 10.3389/fpls.2023.1232588 (PMC10587573; doi:10.3389/fpls.2023.1232588)
Supplement: Supplementary file 1 [file DataSheet_1.zip › Figure S4.PDF]

Alisawi et al. Petunia satellite repeats

Supplementary data Figure S4

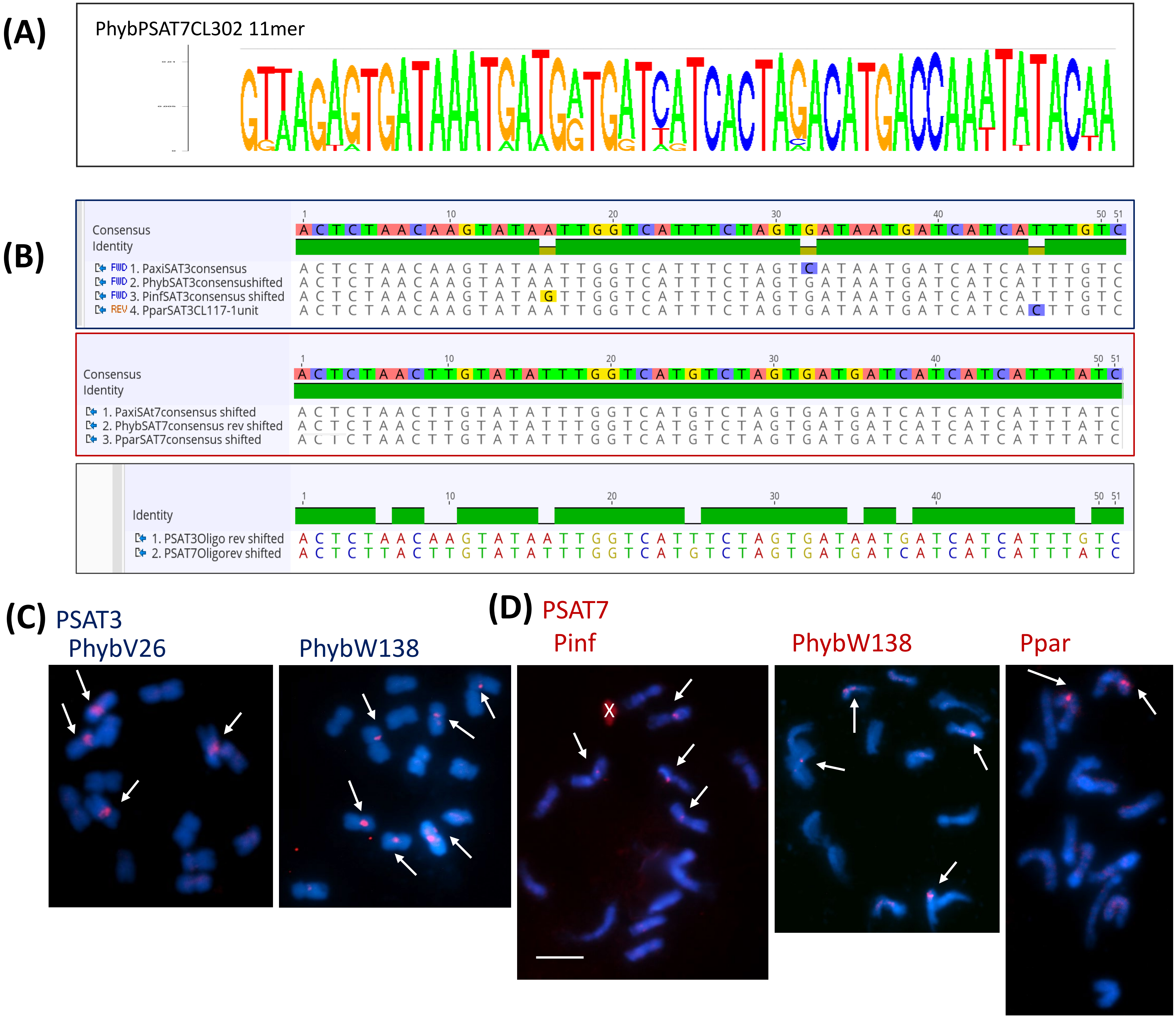

Figure S4: PSAT3 and PSAT7 sequence logos, alignment and chromosomal location

(A) TAREAN Logo of PSAT7 in *P. hybrida* R27.

(B) Sequence alignment of the PSAT 3 (top) and PSAT 7 (middle) and between the two 51 bp monomers showing 8nt differences (84% identity).

(C,D) FISH of PSAT3 (C) and PSAT7 (D) oligonucleotide probe shown in red on blue DAPI chromosomes of *P. hybrida* V26 and W138, *P. inflata* and *P. parodii.* 2-6 signals are visible associated with the centromeres (arrows). Bar = 10µm
