# Supplementary material for: The nature and organization of satellite DNAs in Petunia hybrida, related, and ancestral genomes
Source: Front Plant Sci. 2023 Oct 6;14:1232588. doi: 10.3389/fpls.2023.1232588 (PMC10587573; doi:10.3389/fpls.2023.1232588)
Supplement: Supplementary file 1 [file DataSheet_1.zip › Table S1.docx]

Alisawi et al. Petunia satellite repeats

**Supplementary material**

**Table S1**: **Oligonucleotides and primers used in the study.**

Primers to amplify PSAT1 and PSAT2 from P. axillaris genomic DNA for cloning the two different tandem repeats in cluster CL43. Directly labelled oligonucleotide FISH probes [OligoFISH probes) for six tandem repeat types. They were synthesized with a 5’ attached biotin-11-dUTP [Btn].

| Repeat | Type | Oligonucleotide sequence (5’-3’) | Tm °C | Product or probe length (bp) |
| --- | --- | --- | --- | --- |
| PSAT1 | PCR primers | (Scf160-72F) CCG AAA GCG CAA AC TATC CC  (Scf160-26R) AAA AAG AGG TAG GCG TTG TAA AG | 64.5 | 155 |
| PSAT3 | OligoFISH probe | [Btn]TCACTAGAAATGACCAATTATACTTGTTAGAGTGACAAATGATGATCATTA |  | 51 |
| PSAT4 | OligoFISH probe | [Btn]CCTTTTTGGTATACTGTATACTCTTTCGGTATACCTTGTTATGTTTGGATCGAAG |  | 55 |
| PSAT5 | OligoFISH probe | [Btn]AACATACATAAATATTTGATTGTAGAAAATATTTGAGCCGAAGCGGCCG |  | 49 |
| PSAT6 | OligoFISH probe | [Btn]AAACTGACTCGAAAAGGAAATGATCGCTATCTTTTAGC |  | 38 |
| PSAT7 | OligoFISH probe | [Btn]TGATGATGATCATCACTAGACATGACCAAATATACAAGTAAGAGTGATAAA |  | 51 |
| PSAT8 | OligoFISH probe | [Btn]GGCTACACCATGCGAAGTTCGGGGACGAACTTGCTTTAAAGAAAGGGGGGATGA |  | 54 |
