# Supplementary material for: The nature and organization of satellite DNAs in Petunia hybrida, related, and ancestral genomes
Source: Front Plant Sci. 2023 Oct 6;14:1232588. doi: 10.3389/fpls.2023.1232588 (PMC10587573; doi:10.3389/fpls.2023.1232588)
Supplement: Supplementary file 1 [file DataSheet_1.zip › Table S4.PDF]

Supplementary material

Table S4: Repeat sequences identified as putative satellites in the TAREAN and RepeatExplorer Reports of *P. hybrida* R27 raw reads (*PhybR27*). PhybSATs, consensus sequences, monomer lengths, selected extracted contigs. Repeats that were not found in the TAREAN report as putative satellites, had no consensus sequence; contigs of clusters were searched to find them.

Repeat	Cluster, TAREAN consensus and extract contig sequence	Comments	FISH
**PhybSAT1**<br>168bp<br>monomer	**Cluster CL80**<br>TAREAN consensus (168bp) High confidence putative satellite,0.19% of genome<br>AGCACGAAATACCAGATTTTTCAAAAAACTGAGTACTAGCCCATTTTTCTT**TA**CAA<br>CGCCTACCTCTTTTTCATATGTTCGCTA**TAG**AAAG**T**GCAAACTAT**A**CCTTTTTATAG<br>CATTTCTTGACAAAATTTTGAATTTTCATTTTTTGAACAGTCCGCACCTTCAGAA<br>CL80 Contig27 extraction (504bp, with three 168bp monomers)<br>GATTTTTCAAAAAACTGAGTACTAGCCCATTTTTCTT**TA**CAA<br>CGCCTACCTCTTTTTCATATGTTCGCTA**TAG**AAAG**T**GCAAACTAT**A**CCTTTTTATA<br>GCATTTCTTGACAAAATTTTGAATTTTCATTTTTTGAACAGTCCGCACCTTCAGAA<br>AGCACGAAATACCAGATTTTTCAAAAAACTGAGTACTAGCCCATTTTTCTT**TA**CAA<br>CGCCTACCTCTTTTTCATATGTTCGCTA**TAG**AAAG**T**GCAAACTAT**A**CCTTTTTATA<br>GCATTTCTTGACAAAATTTTGAATTTTCATTTTTTGAACAGTCCGCACCTTCAGAA<br>AGCACGAAATACCAGATTTTTCAAAAAACTGAGTACTAGCCCATTTTTCTT**TA**CAA<br>CGCCTACCTCTTTTTCATATGTTCGCTA**TAG**AAAG**T**GCAAACTA**TA**CCTTTTTATA<br>GCATTTCTTGACAAAATTTTGAATTTTCATTTTTTGAACAGTCCGCACCTTCAGAA<br>AGCACGAAATACCA	Forward Primer (Scf160-72F: CCGAAAGCGCAAACTATCCC) underlined;<br>Reverse Primer (Scf160-26R: AAAAAGAGGTAG GCGTTGAAG) underlined with some mismatches in bold<br>Reverse comp<br>CTTCAACGCCTACCTCTTTTT	FISH: on all telomeres except short arm of Chrs II and III.<br>In W138, the short arm of Chr III has a signal.

**PhybSAT3**<br>Monomer<br>51bp	**Cluster 101**<br>TAREAN consensus (51bp) Low confidence putative satellite,0.15% of the genome<br>TAACAAGTATAATTGGTCATTTCTAGTGATAATGATCATCATTTGTCACTC<br>CL101 Contig37 extraction (823bp, reverse compliment)<br>C<br>TAACAAGTATAGTCGGTCATTTCTAGTTATAATGATCATCATTTGTCACTC<br>TTACAAGTATAGTGGGTCATTTCTAGTCATAATGATCATCATTTCTCACT**A**<br>TAACAAGTATAATTGGTCATTTCTAGTGATAATGATCATCATTTGTCACTC<br>TAACAAGTACAGTGGGTCATTTCTAGTGACAATGATCATCACTTGTCACTC<br>TTACAAGTGTAATTGGCCATTTCTAGTGATAATGATCATCATTTGTCA**GTT**<br>TAACTACCATAATTGGTCATTT...............ATCATTTGTCACTC<br>TTACAAGTATAGTGGGTTATTTCTAGTGATAATGATCATCACTTATCACTC<br>TAACAAGTGTAATTGGCCATTTCTACTCATAATGATCATCATTTGTCACTC<br>TAACAAGTATAATTGGTCATTTCTAGTGATAATGATCATCATTTGTCACTC<br>TTACAAGTGTAATTGGCTATTTGTAGTGACAATGATCATCATTTCTCACT**A**<br>TAACAAGTATAATTGGTCATTTCTAGTGATAATGATCATCATTTGTCACTC<br>TAACAAGTATAATGGGTCATTTCTAGTGATAATGATCATCAATTCTCAGTC<br>TAACTACTCTATTTGGACTTTTGTGGTGAA$_{AG}$TTGATGA..ATTTATCACTC<br>TTACAAGTATAATTGGTCACTTATAGTGGTATT.ATCATC$_{ATAATT}$TGTCACTC<br>TAATTACTCTATTTGG<br>GAAACAATACGATGAAGATTAAAACAATTAATGACCGCACTCAGAACGACTTATTAA<br>ATGTGACAAACATCAATTGGATGTTCGGAACCACTCGATGATGGATGCA<br>Oligo FISH probe (Reverse):<br>TAATGATCATCATTTGTCACTC<br>TAACAAGTATAATTGGTCATTTCTAGT**G**A	Monomer of 51bp (highlighted in yellow and grey),<br>fourteen perfect and less perfect monomers are present in the extracted contig	FISH: 4-6 signals probably on Chr III and another small metacentric chromosome

**PhybSAT4**<br>Monomer:<br>113bp	**Cluster CL116**<br>TAREAN consensus (113bp) Low confidence putative satellite,0.093% of genome<br>AACTGAAATATTT.ATT**C**GCTCGGTAG**C**ATC**G**CACACTT**G**GATCCAAACA**C**AA**A**A**G**G<br>GTATA<br>CC**AG**AAGAGTATACAGTATACCAAAA**G**GGTATA**C**T**T**TGTTCAAAA**C**AACAA**A**<br>CL116 Contig7 extraction (250bp)<br>TTTTTATTTTTTTAATTTATTTTCTTTCTCCGTAATCTTATTATACTCAATTTTTTAT<br>TTTTTTTCTTTCTCCAAAAAGGCATACTTTGTTCACAAACACAAA<br>AACTGAAATATTTTATT**T**GCTCGATAG**T**ATC**T**CACACTT**TT**ATCCAAACA**T**AA**C**A**A**G<br>GTATACC**GA**AAGAGTATACAGTATACCAAAA**A**GGTATA**T**T**C**TGTTCAAAA**T**AACAA**T**<br>AATTGAAATAATCATTTACTCGATAACATCACAC<br>OligoFISH probe (55bp) reverse<br>CTT**C**GATCCAAACA**T**AACAAG<br>GTATACCGAAAGAGTATACAGTATACCAAAAAGG	TAREAN consensus in yellow, oligoFISH probe underlined	Weak and dispersed signal<br>(

**PhybSAT5**<br>Monomer<br>78bp	Cluster CL114<br>No TAREAN consensus<br>CL114 Contig5 extraction (1166bp, reverse)<br>CGGGTGAGGAGGTTGTTGTGCCGTTTCCCTCGCCGTTTTGAGGTGCCAATACCGTCC<br>GGCCAGCGGACACACCCCGACCGGCCGACACGCTTGGTGGCCAACGTAACGTTGCAC<br>GGCCAAGGCCATGCGTGCTATCGTTGCCCACGCTGCGTGCCAGCATGGCCGACATGT<br>GCCTGCCTTAGGCCTGCTGCAGGCGCCCCAAGCCTGCTCAACACCTCACCCCTCCCT<br>ATATATGTCCTTAAAAAAAAAACCCAAGTCTCAGGAGTAGACATGCTTTGGCCAGAG<br>AATCATACTGGACGTGTACAACACCAATGTGCACAATCTCAAGTACAACCACCCCAA<br>AGCGTTCAAAATGACTTCCAAACTATGAAAATAGTATAAAATATTGTAAATTATATT<br>TTTTTTATAAAATACATAAATAATATTAAATAAATATAATAAAATACCAAAAAATTT<br>CAAAAAAATATTATAATTTATTATAGAACCACAGAAATGATTGTAGCATGATTAAAT<br>TATGTAAATTTAGAAAATAGATTAATATTTAAATAATAAAAAAGTAATAAAAAGTAT<br>TTAAAATACATAAATAATAATATAAATATTAAATAAATATAATAAAATACCAAAAAT<br>CACAAAAAAGTATTATAATTTATTATAGAAAGCCACAAATGATTGTAGCATGATTGT<br>ATTATGTAAATTTAGAAAATAAATTAATATTTAAATAATAAAAAAGTAATAAATAAA<br>TAAAAATATTATTTTGGTGGAGAAAAATATCAAGGGCATCGGGTAAGTACACGATAC<br>ACGAAATGAGTATGAAAAGACATAAATGAGTAGACACCAATAGTCAAAAGAGATGCA<br>ATGATTTAGTTTGGCGGTGTTTCAGTATAAGAAACGTTTGCACCACATTATATATCG<br>GTACGTACTCAATCGTGCACAACGGTACATATCGAACTTCTTTTTGGAACATAGAGA<br>GACCGTGCACTCGGCCACTA<br>CGGCTTAGTCCCTCGGCCGCTTCGGCTCAAATATTTTCTACAATCAAATATTTATGT<br>ATGTTATGCATCAAAAAGCTT<br>CGGCTTAGTCCCTCGGCCGCTTCGGCTCAAATATTTTCTACAATCAAATATTTATGT<br>ATGTTATGCATCAAAAAGCTT<br>CGGCTTAGTCGCTCGGCCGCT<br>oligoFISH probe revers (49bp)<br>CGGCCGCTTCGGCTCAAATATTTTCTACAATCAAATATTTATGTATGTT	The 78bp repeat unit (highlighted in yellow and grey) together with the OligoFISH probe (underlined) included twice towards the end. Leading stretch has several AT rich motifs	FISH<br>4-6 signals near centromere<br>Probably Chr II and III and a larger metacentric chromosome

**PhybSAT6**<br><br>78bp monomer with 2 subunits A and A’	Cluster CL145<br>No TAREAN consensus<br>CL145 Contig7 (616bp, reverse)<br>TCGATCATTTCCTTTTCGA GTCAGTTTTC<br>GCTCAAAGATAGCG TCATTTCTTTTCCATGTCAAGATCT (39bp)<br>GCTAAAAGATAGCGATCATTTCCTTTTCGAGTCAG TTC<br>GCTCAAAGATAGCG TCATTTCCTTTCCATGTCAAGATCT (39bp)<br><u>GCTAAAAGATAGCGATCATTTCCTTTTCGAGTCAGTTT</u>C (39bp)<br>GCTCAAAGATAGCG TCATTTCCTTTCCATGTCAAGATCT (39bp)<br>GCTAAAAGATAGCGATCATTTCCTTTTCGAGTCAGTTTC (39bp)<br>GCTCAAAGATAGCG TCATTTCCTTTCCATGTCAAGATCT (39bp)<br>GCTAAAAGATAGCGATCATTTCCTTTTCGAGTCAGTTTC (39bp)<br>GCTCAAAGATAGCG TCATTTCCTTTCCATGTCAAGATCT (39bp)<br>GCTAAAAGATAGCGATCATTTTCCTTTC AAGTCAGTTTT<br>GCTAGAAGATAGCACTCACATTTAGTTCAAGTCATTTGC<br>GCTCAAAGATAGCAACCACTTCCTTTTCAAGTTAGTCCT<br>GCTCGAAGATAGCAACCAT<br>CCCCAGCTTCAAGTCAAGACCTGGTTGAAACAGATGCGACCATTTCAAGTTTCAGAT<br>GGTGCCTGCCCATACAACGGGTGACGGAGTTTGTCTTTCAAGCC<br>Oligo FISH probe (38bp) Reverse<br>GCTAAAAGATAGCGATCATTTCCTTTTCGAGTCAGTTT	78bp monomer with two subunits of A (39bp) and A’ (39bp)<br><br>Oligo FISH probe (underlined) is the 38bp of the A’ repeat	FISH: 2-4 signals near centromere, and some look clearly like satellited chr. So II or III

**PhybSAT7**<br>51 bp<br>monomer	Cluster CL302:<br>TAREAN consensus (51bp)<br>GTTAGAGTGATAAATGATGATGATCATCACTAGACATGACCAAATATACAA<br>Reverse<br>TTGTATATTTGGTCATGTCTAGTGATGATCATCATCATTTATCACTCTAAC<br>CL302 Contig1 extraction (374bp) including six 51 bp monomers<br>.......................**ATTGTTAT**CAT.CATTTATCACTCT**AT**C<br>TTGTA.A.TTGGTCAAGTCTAGTGATGATCACAT.CATTTGTCACTCT**AA**C<br>TTGTATATTTGGTCATGTCTAGTGATGATCATCATCATTTATCACTCT**TA**C<br>TTGTATATTTGGTCATGTCTAGTGATGATCATCATCATTTATCACTCT**TA**C<br>TTGTATATTTGGTCATGTCTAGTGATGATCATCATCATTTATCACTCT**AC**C<br>TTGTATATTTGGTCATGTCTAGTGATGATCATCATCATTTATCACTCT**TA**C<br>TTGTATATTTGGTCATGTCTAGTGAT**T**ATCACCATCATTTATCACTAT**AA**C<br>TAGTATAATTG**A**TCAT**T**TGTAGT**T**AT**A**ATCACCA**A**CA**G**TTCTCC<br>Oligo FISH probe (51bp) Reverse<br>.....................................TTTATCACTCTTAC<br>TTGTATATTTGGTCATGTCTAGTGATGATCATCATCA	Monomer of 51bp in yellow and grey, Oligo FISH probe (rev) underlined<br>Mismatches in bold	FISH: One pair of signals maybe another weak in some metacentric chromosomes in some accessions

**PhybSAT8**<br>Monomer<br>297bp<br><br>CL424Contig2	**Cluster CL424**<br>TAREAN consensus (297bp)<br>CCCTTTCTTTAAAGCAAGTTCGTCCCCGAACTTCGCATGGTGTAGCCAGGAATCGAA<br>CCCGGGTTGGCTCTGATACCAAACTTTAAGAGTGCACTGCAACCATTTCTACCAAAA<br>GCCATAGCTGATGGTAGAGGCGCAACGCAACTCTTATACCGCCGGATCAACCCCAAA<br>TCGAGGTCCGTCCCTGACACCACCATGACCACCCCGAGCAACGCAACTCCTCCAGGG<br>GGTGGACCCCACTACGGGCGGCTCCTTATGATGCGGGCCCAATCCTTGATAGCCCAG<br>CTGCATCATCCC<br>Reverse<br>GGGATGATGCAGCTGGGCTATCAAGGATTGGGCCCGCATCATAAGGAGCCGCCCGTA<br>GTGGGGTCCACCCCCTGGAGGAGTTGCGTTGCTCGGGGTGGTCATGGTGGTGTCAGG<br>GACGGACCTCGATTTGGGGTTGATCCGGCGGTATAAGAGTTGCGTTGCGCCTCTACC<br>ATCAGCTATGGCTTTTGGTAGAAATGGTTGCAGTGCACTCTTAAAGTTTGGTATCAG<br>AGCCAACCCGGGTTCGATTCCTGGCTACACCATGCGAAGTTCGGGGACGAACTTGCT<br>TTAAAGAAAGGG<br>CL424 contig2 extraction (394bp)<br>.CCGTGATGCAGCTGGGCTATCA<br>**A**GGAT**TGG**GC**C.**C**G**GGGCTATCAAGGATTGGGCCCGCATCATAAGGAGCCGCCCGTA<br>GTGGGGTCCACCCCCTGGAGGAGTTGCGTTGCTCGGGGTGGTCATGGTGGTGTCAGG<br>GACGGACCTCGATTTGGGGTTGATCCGGCGGTATAAGAGTTGCGTTGCGCCTCTACC<br>ATCAGCTATGGCTTTTGGTAGAAATGGTTGCAGTGCACTCTTAAAGTTTGGTATCAG<br>AGCCAACCCGGGTTCGATTCCTGGCTACACCATGCGAAGTTCGGGGACGAACTTGCT<br>TTAAAGAAAGGGGGGATGATGCAGGGCCGCTATCAAGGATTGGGCCCGGG<br>GCTATCAAGGATTGGGCCCACATCATAAGGAGCCGCCC<br>Oligo FISH probe (54bp)<br>GGCTACACCATGCGAAGTTCGGGGACGAACTTGCT<br>TTAAAGAAAGGGGGGATGA	Pinf has PSAT in a TAREAN cluster with a 294bp consensus, but Paxi has not.<br>TAREAN consensus in yellow.<br><br>Includes the TAREAN consensus (yellow, but with mismatches at the beginning)<br>Oligo FISH probe underlined<br>Mismatches in bold	NO FISH signal
