# Supplementary material for: The nature and organization of satellite DNAs in Petunia hybrida, related, and ancestral genomes
Source: Front Plant Sci. 2023 Oct 6;14:1232588. doi: 10.3389/fpls.2023.1232588 (PMC10587573; doi:10.3389/fpls.2023.1232588)
Supplement: Supplementary file 1 [file DataSheet_1.zip › Table S5.PDF]

Table S5: Repeat sequences identified as putative satellites in the TAREAN and Repeat Explorer Reports of *P. axillaris* N raw reads (*PaxiN*). PaxiSATs, consensus sequences, monomer lengths, selected extracted contigs. Repeats that were not found in the TAREAN report as putative satellites, had no consensus sequence; contigs of clusters were searched to find them.

Repeat	Cluster, TAREAN consensus and extract contig sequence	Comments	FISH
**PaxiSAT1**<br><br>168bp monomer	**Cluster CL107**<br>TAREAN consensus (168bp), high confidence putative satellite, 0.2% of the genome<br>GTTCAAAAAATGAAAATTCAAAATTTTGTCAAGAAATGCTATAAAAAGG**T**ATAGT<br>TTGC**A**CTTTC**TA**TAGCGAACATATGAAAAAGAGGTAGGCGTTGT**A**AAGAAAAATG<br>GGCTAGTA**C**TCAGTTTTTTGAAAAATCTGGTATTTCGTGCATTCTGAAGGTGCGG<br>ACT<br>CL107Contig27 (504bp with three 168p monomers)<br>TTCAAAATTTTGTCAAGAAATGCTATAAAAAGG**T**ATAGT<br>TTGC**A**CTTTC**TA**TAGCGAACATATGAAAAAGAGGTAGGCGTTGT**A**AAGAAAAATG<br>GGCTAGTACTCAGTTTTTTGAAAAATCTGGTATTTCGTGCATTCTGAAGGTGCGG<br>ACT<br>GTTCAAAAAATGAAAATTCAAAATTTTGTCAAGAAATGCTATAAAAAGG**T**ATAGT<br>TTGC**A**CTTTC**TA**TAGCGAACATATGAAAAAGAGGTAGGCGTTGT**A**AAGAAAAATG<br>GGCTAGTACTCAGTTTTTTGAAAAATCTGGTATTTCGTGCATTCTGAAGGTGCGG<br>ACT<br>GTTCAAAAAATGAAAATTCAAAATTTTGTCAAGAAATGCTATAAAAAGG**T**ATAGT<br>TTGC**A**CTTTC**TA**TAGCGAACATATGAAAAAGAGGTAGGCGTTGT**A**AAGAAAAATG<br>GGCTAGTACTCAGTTTTTTGAAAAATCTGGTATTTCGTGCATTCTGAAGGTGCGG<br>ACT<br>GTTCAAAAAATGAAAA<br>PaxiSAT1 (155bp) clone insert for comparison<br>AAAAAGAGGTAGGCGTTGT**A**AAGAAAAATGGGCTAGTA**T**TCAGTTTTTTGAAAAATCCGGT<br>ATTTCGTGCATTCTGAAGCTGCGGACTGTTCAAAAAATGTAAATTCAAAATTTTGTCAAGA<br>AATGCTATAAAAAGGGATAGTTTGCGCTTTCGG	Primers used for cloning the 157bp part:<br>Scf160-72F:<br>CCGAAAGCGCAAACTATCCC (underlined)<br>Scf160-26R:<br>AAAAAGAGGTAGGCGTTGAAG (double underlined)<br>Mismatches in bold. The cluster monomer has 13 bp (highlighted in cyan) more than the 155bp clone.<br>Reverse and forward primer have mismatches.	At telomeres of all chromosomes, except the short arms of chromosomes II and III that are unequal armed and carry the 45S rDNA on the short arm close to the telomere.<br>Chr II also has a 5SrDNA site on the short arm; the other 5S rDNA chromosome (chromosome IV) has signals on both ends.

**PaxiSAT3**<br><br>51bp monomer:	Cluster CL58<br>TAREAN consensus (51bp), high confidence putative satellite<br>0.29% of genome<br>ACTCTAACAAGTATAATTGGTCATTTCTAGT**C**ATAATGATCATCATTTGTC<br>CL58, Contig 19 (408bp, with eight 51bp monomers)<br>CTAACAAGTATAATTGGTCATTTCTAGT**C**ATAATGATCATCATTTGTC<br>ACTCTAACAAGTATAATTGGTCATTTCTAGT**G**ATAATGATCATCATTTGTC<br>ACTCTAACAAGTATAATTGGTCATTTCTAGT**C**ATAATGATCATCATTTGTC<br>ACTCTAACAAGTATAATTGGTCATTTCTAGT**G**ATAATGATCATCATTTGTC<br>ACTCTAACAAGTATAATTGGTCATTTCTAGT**C**ATAATGATCATCATTTGTC<br>ACTCTAACAAGTATAATTGGTCATTTCTAGT**G**ATAATGATCATCATTTGTC<br>ACTCTAACAAGTATAATTGGTCATTTCTAGT**C**ATAATGATCATCATTTGTC<br>ACTCTAACAAGTATAATTGGTCATTTCTAGT**G**ATAATGATCATCATTTGTC<br>ACT<br>OligoFISH probe Reverse:<br>TAATGATCATCATTTGTC<br>ACTCTAACAAGTATAATTGGTCATTTCTAGT**G**A	Eight 51bp monomer (in alternate grey and yellow highlight) one alternate mismatch (bold)<br>OligoFISH probe (rev) underlined in first occurrence only	**FISH**<br>Fig S3I<br>Six signals:<br>Strong signal near cent of an unequalled arm chr (II or III)<br>A pair of strong and a pair of weak signals mid arm on two meta centric chromosomes (chr IV-VI)

**PaxiSAT4**<br><br>Monomer: 113bp	**Cluster CL202**<br>TAREAN consensus (113bp): low confidence putative satellite, 0.092% of the genome<br>...........................AACTGAAATATTT ATTCGCTCGGTAG**C**A<br>TC**G**CACACTT**TG**ATCCAAACA**C**AAAAGGGTATACCAGAAGAGTATACAGTATACCAA<br>AAGGGTATACTTTGTTCAAAACAACAAA<br>CL202Contig33 extraction (154bp)<br>AAAGGCATACTTTGTTCACAAACACAAAAACTGAAATATTTTATTTGCTCGATAG**T**A<br>TC**T**CACACTT**TT**ATCCAAACATAACAAGGTATACCGAAAGAGTATACAGTATACCAA<br>AAAGGTATATTCTGTTCAAAA**T**AACAA**T**AACTGAAATACT<br>OligoFISH probe (55bp) Reverse:<br>CTT**CG**ATCCAAACATAACAAGGTATACCGAAAGAGTATACAGTATACCAA<br>AAAGG	113bp consensus in yellow OligoFISH probe underlined, mismatches in bold.	Dispersed FISH signal

 PaxiSAT5<br><br>78bp<br>monomer	Cluster 159<br>No TAREAN consensus<br>CL159 Contig 7 extraction (1044bp)<br>ACTAAGCCAAAGTAAGCGGAAGCGGCCGAGGGACTAAGCCAAAGCGGCCGAACTACTAAGC<br>CGAAGTAGGCGGAAGCGGCCGAGATACTAAGCCAAAGTAAGCGGAAGCGGCCGAGGTACTA<br>AGCCAAAGCGGCCGGACTACTAAGCCGGTGCTTCG<br>**TGATG**TTTTGATGCATAACATACATAAATATTTGATTGTAG**G**AAATATTTGAGCC<br>GAAGCGGCCGAGCGACTAAGCCG<br>AAGCTTTTTGATGCATAACATACATAAATATTTGATTGTAG**G**AAATATTTGAGCC<br>GAAGCGGCCGAGGGACTAAGCCG<br>TAGTGGCCGAGTGCACGGTCTCTCTATGTTCCAAAAAGAAGTTCGATATGTACCGTTGTGC<br>ACGATTGAGTACGTACCGATATATAATGTGGTGCAAACGTTTCTTATACTGAAACACCGCC<br>AAACTAAAACATTGCATCTCTTTTGACTATTGGTGTCTACTCATTTATGTCTTTTCATACT<br>CATTTCGTGTATCGTGTACTTACCCGATGCCCTTGATATTTTTCTCCACCAAAATAATATT<br>TTTATTTATTTATTACTTTTTTATTATTTAAATATTTATTTATTTTCTAAATTTACATAAT<br>ACAATCATGCTACAATCATTTGTGGCTTTCTATAATAAATTATAATACTTTTTTGTGATTT<br>TTGGTATTTTATTATATTTATTTAATATTTATATTATTATTTATGTATTTTAAATACTTTT<br>TATTACTTTTTTATTATTTAAATATTAATCTATTTTCTAAATTTACATAATTTAATCATAC<br>TACAATCATTTGTGTGGTTGTTGAAGTCATTTATAATATTTTTTTGTATTTTATTATATTT<br>ATTTAATATTTATATTATTATTTAGGTATTTTATAAAAAAAAATATAATTTACAATATTTT<br>ATACTATTTTCATAGTTTGGAAGTCATTTTGAACGCTTTGGGGTGGTTGTACTTGAGATTG<br>TGCACATTGGTGTTGTACACGTCCAGTATGATTCTCTGGCCAAAGCATGTCTACTCCTGA<br>oligoFISH probe (49bp)<br>AACATACATAAATATTTGATTGTAGAAAATATTTGAGCC<br>GAAGCGGCCG	Two copies of a 78bp unit found (yellow and grey)<br>mismatches in bold<br>Remaining part has AT rich motifs<br><br>OligoFISH probe underlined	FISH<br>Strong signal at the end of one pair (unequalled arm chromosome, probably Chr III)<br>6 signals near the centromeres of other chromosomes

**PaxiSAT6**<br><br>77 bp monomer with two subunits A and A’	Cluster 168<br>No TAREAN report<br>CL168 Contig 18 extraction (188bp):<br>CTTTTC**GA**GTCA**GTT**TC<br>GCT**C**AAAGATAGCG TCATTTCCTTT C**AT**GTCA**AGA**TC**T** (38bp)<br>GCT**A**AAAGATAGCGATCATTTCCTTTTC**GA**GTCA**GTT**TC (39bp)<br>GCT**C**AAAGATAGCG TCATTTCCTTT C**AT**GTCA**AGA**TC**T** (38bp)<br>GCT**A**AAAGATAGCGATCATTTCCTTTTTG**AA**GTCA**GTT**TC (40bp)<br>**GCT**CAAAGATAGCG TC<br>Oligo FISH probe (38bp)<br>reverse<br>GCTAAAAGATAGCGATCATTTCCTTTTCGAGTCAGTTT	Monomer unit of 77bp made up of A(38bp)+A’(39bp)<br>OligoFISH probe underlined in subunit A’ repeat	**FISH**<br>One pair of signals at the centromere of an equal armed chr.
PaxiSAT7<br>51bp monomer	Cluster CL290<br>TATREAN consensus<br>CACTCTAACTTGTATATTTGGTCATGTCTAGTGATGATCATCATCATTTAT<br><br>CL290 Contig 23 extraction (357bp) with eight 51bp monomers<br>ATCATCA<br>TTTATCACTCTTACTTGTATATTTGGTCATGTCTAGTGATGATCATCATCA<br>TTTATCACTCTTACTTGTATATTTGGTCATGTCTAGTGATGATCATCATCA<br>TTTATCACTCTTACTTGTATATTTGGTCATGTCTAGTGATGATCATCATCA<br>TTTATCACTCTTACTTGTATATTTGGTCATGTCTAGT**GATGATCATCATCA**<br>**T**TTATCACTCTTACTTGTATATTTGGTCATGTCTAGTGATGATCATCATCA<br>TTTATCACTCTTACTTGTATATTTGGTCATGTCTAGTGATGATCATCATCA<br>TTTATCACTCTTACTTGTATATTTGGTCATGTCTAGTGATGATC<br>Oligo FISH probe (51bp) reverse<br>TTTATCACTCTTACTTGTATATTTGGTCATGTCTAGTGATGATCATCATCA	Monomer is 51bp and was used as oligo FISH probe<br>PaxiSAT7 has high homology to PaxiSAT3	FISH (Fig2K)<br>5-6 signals;<br>Strong signal mid arm of a large equal armed chr.<br>And centromeric on an unequal armed chr (II or III)

**PaxiSAT8**<br><br>192bp monomer	Cluster 373<br>NO TAREAN consensus<br>CL373 Contig 2 extraction(450bp) Reverse<br>GCCTCTACCATCAGCTATGGCTTTTGGTAGAAATGGTTGCAGTGCC<br>TCTCTTAAAGTTTGGTATCAGAGCCAACCCGGGTTCGATTCCT<br>GGCTACACCATGCGAAGTTCGGGGACGAACTTGCTTTAAAGAAAGGGGGGATGA<br>TGCAGCTGGGCTATCAA**G**GATTGG**GCC**C<br>GGGGCTATCAAGGATTGGGCCCGCATCATAAG<br>GAGCCGCCCGTAGTGGGGTCCACCCCCTGGAGGAGTTGCGTTGCTCGGGGTGGT<br>CATGGTGGTGTCAGGGACGGACCTCGATTTGGGGTTGATCCGGCGGTATAAGAG<br>TTGCGTTGCGCCTCTACCATCAGCTATGGCTTTTGGTAGAAATGGTTGCAGTGC<br>TCTCTTAAAGTTTGGTATCAGAGCCAACCCGGGTTCGATTCCC<br>GGCTACACCATGCGAAGTTCGGGGACGAACTTGCTTTAAA**GA**<br>Oligo FISH probe (54bp)<br>GGCTACACCATGCGAAGTTCGGGGACGAACTTGCTTTAAAGAAAGGGGGGATGA	At the beginning, there is a 143bp part homologous to the consensus of Phyb, Pinf and Ppar (grey) containing the OligoFISH probe (underlined), a 2nd 292bp (yellow) with some mismatches (bold) does not contain the full OligoFISH probe (underlined).	No clear FISH signal
