# Supplementary material for: The nature and organization of satellite DNAs in Petunia hybrida, related, and ancestral genomes
Source: Front Plant Sci. 2023 Oct 6;14:1232588. doi: 10.3389/fpls.2023.1232588 (PMC10587573; doi:10.3389/fpls.2023.1232588)
Supplement: Supplementary file 1 [file DataSheet_1.zip › Figure S6.PDF]

Alisawi et al. Petunia satellite repeats

Supplementary data Figure S6

(A)

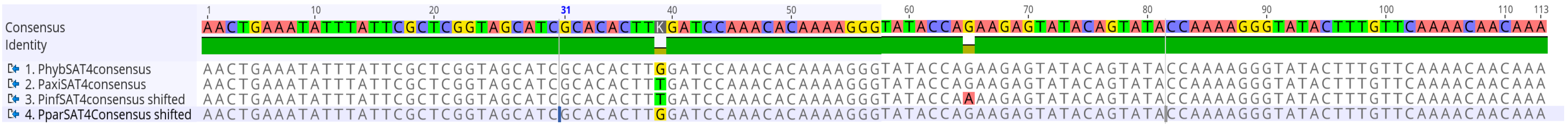

(B)

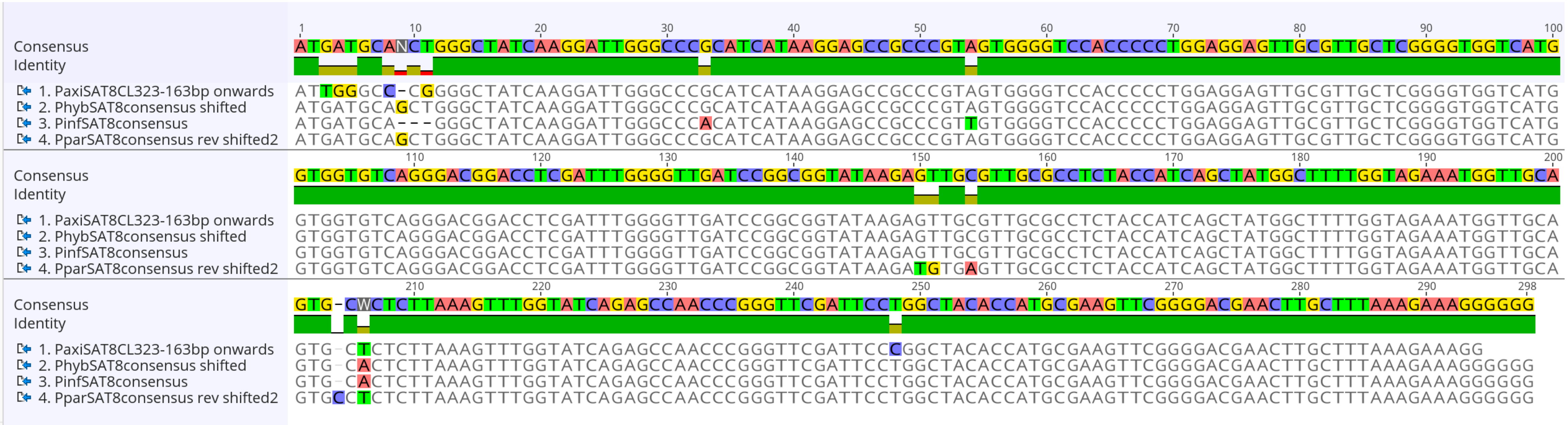

(C) PSAT4 Phyb RdC PSAT8 Paxi

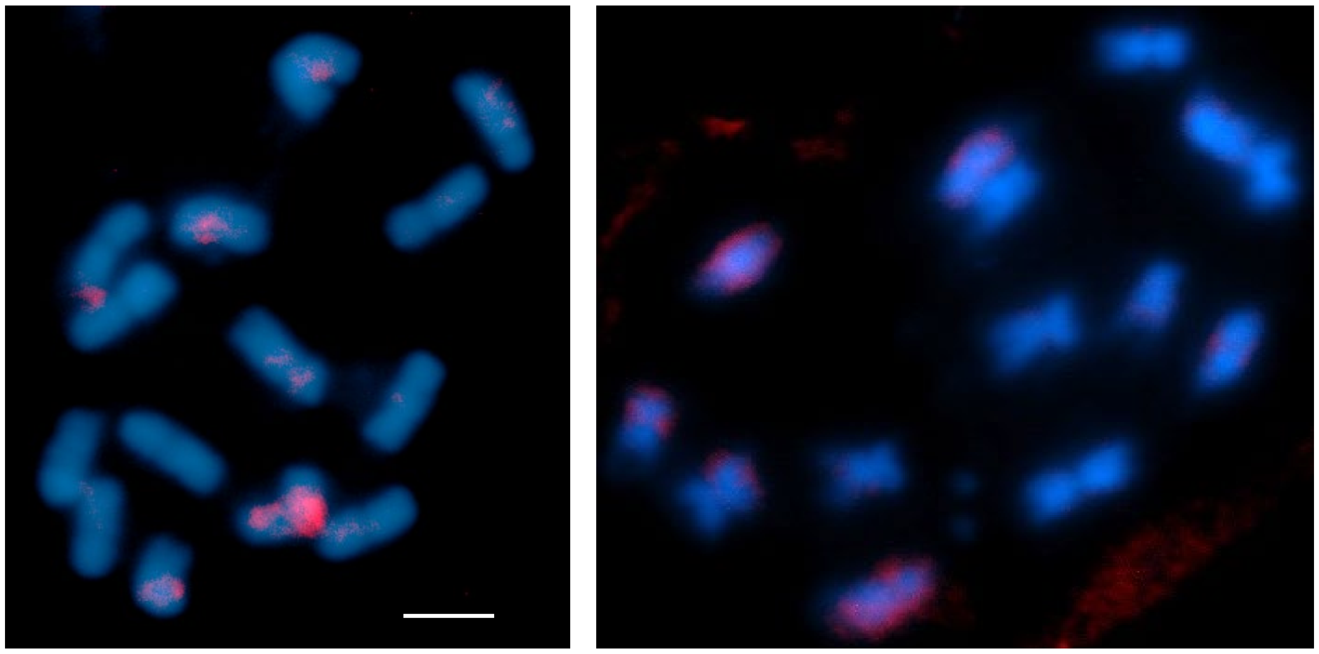

Figure S6: PSAT4 and PSAT8 sequence alignments and chromosomal location

(A) Sequence alignment of the PSAT 4 with a 113bp monomer

(B) Sequence alignment of the PSAT 8 with a 292-298 bp monomer

(C) FISH of PSAT4 oligonucleotide probe in *P. hybrida* RdC (left) and PSAT8 oligonucleotide probe *P. axillaris* (right). Weak to dispersed signal is seen Bar = 10µm
