# Supplementary material for: The nature and organization of satellite DNAs in Petunia hybrida, related, and ancestral genomes
Source: Front Plant Sci. 2023 Oct 6;14:1232588. doi: 10.3389/fpls.2023.1232588 (PMC10587573; doi:10.3389/fpls.2023.1232588)
Supplement: Supplementary file 1 [file DataSheet_1.zip › Figure S5.PDF]

Alisawi et al. Petunia satellite repeats

Supplementary data Figure S5

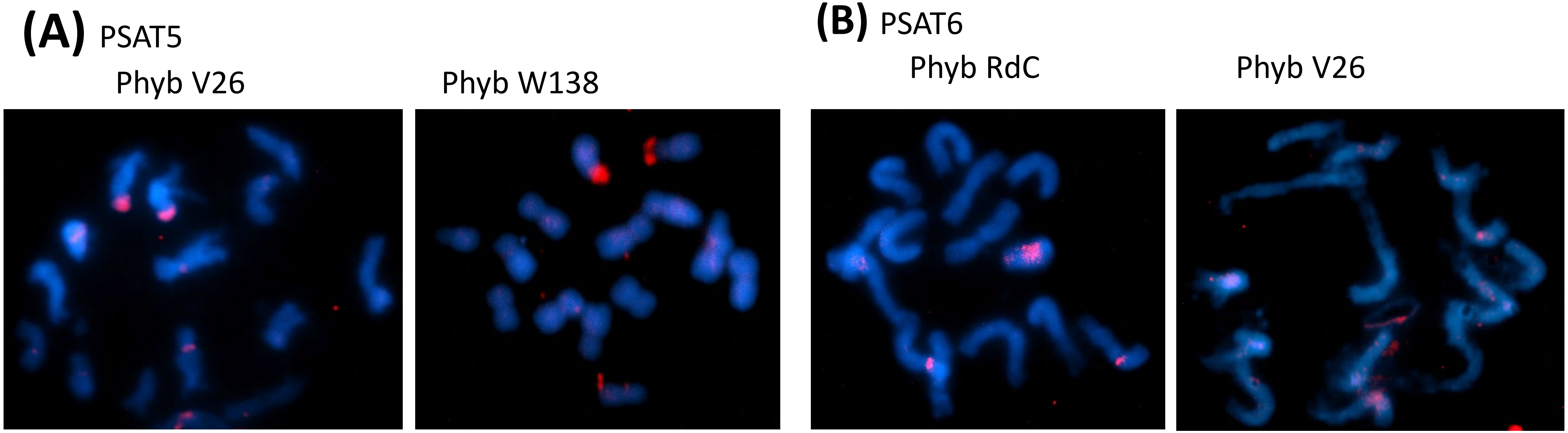

Figure S5: PSAT5 and PSAT6 additional FISH images for *P. hybrida*

Probes are shown in red and chromosomes ares tained with DAPI (blue)

(A) PSAT5 oligonucleotide probe in *P. hybrida* V26 and W138. a pair of strong signals are seen at the end of the short arm of Chr III.

(B) PSAT6 oligonucleotide probe in *P. hybrida RdC* and V26; 2-4 signals are seen near the centromeres.

Bar = 10µm
