# Supplementary material for: The nature and organization of satellite DNAs in Petunia hybrida, related, and ancestral genomes
Source: Front Plant Sci. 2023 Oct 6;14:1232588. doi: 10.3389/fpls.2023.1232588 (PMC10587573; doi:10.3389/fpls.2023.1232588)
Supplement: Supplementary file 1 [file DataSheet_1.zip › Figure S3.PDF]

Alisawi et al. Petunia satellite repeats

Supplementary data Figure S3

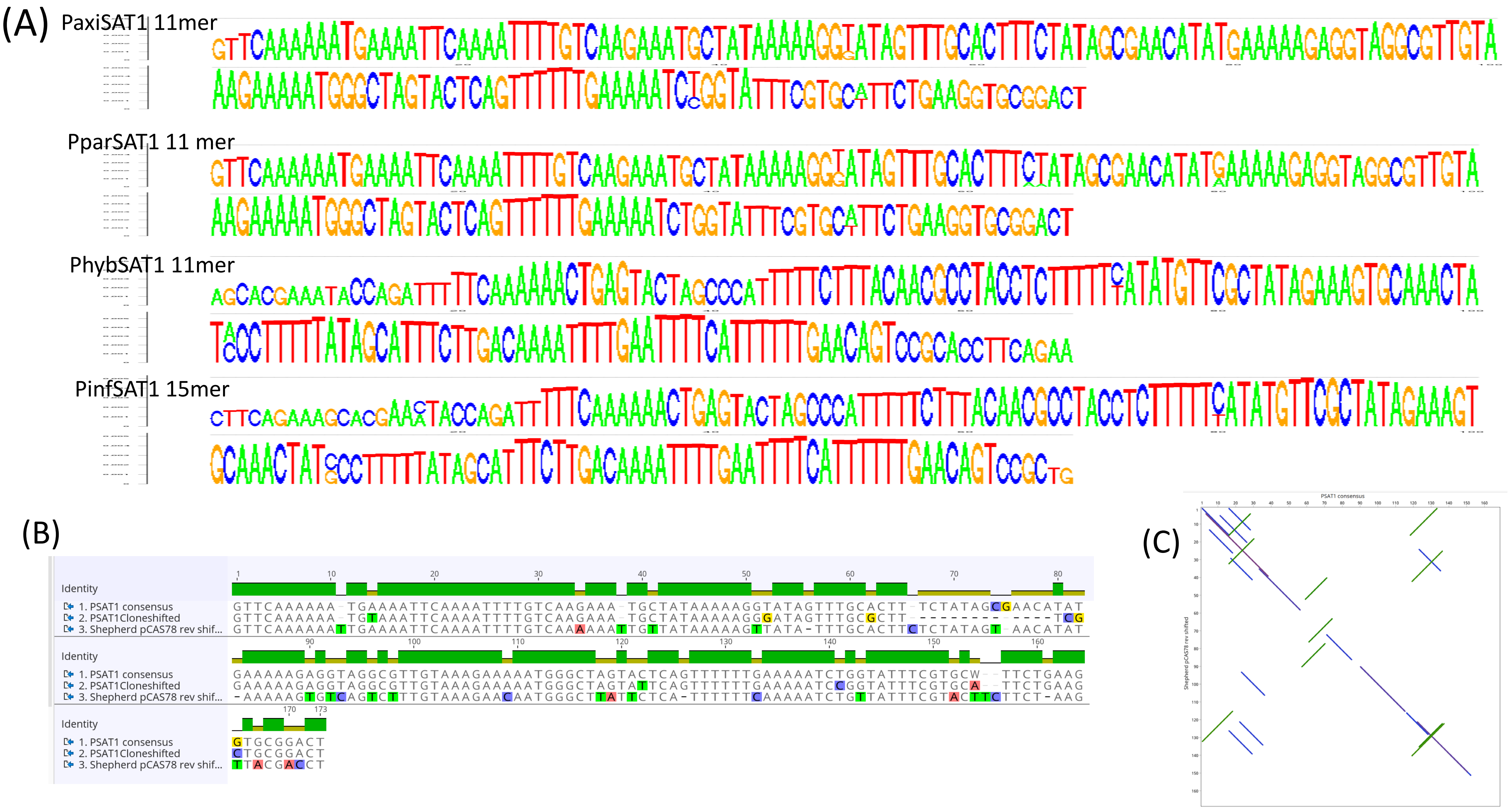

Figure S3: PSAT1 Sequence logos, alignments and comparisons

(A) PSAT1 TAREAN Logos. Very little sequence divergence is found within and between species. Note that the Logos for *P. axillaris* and *P. parodii* are presented in the same direction and start with the same bp of the monomer. *P. hybrida* and *P. inflata* are presented in the reverse compliment and start at different positions.

(B) Sequence comparison and (C) dotblot of PSAT1 consensus, PaxiSAT1Clone and the pCAS78 of Shepherd et al 1990).

Alisawi et al. Petunia satellite repeats

Supplementary data Figure S3 cont.

(D)

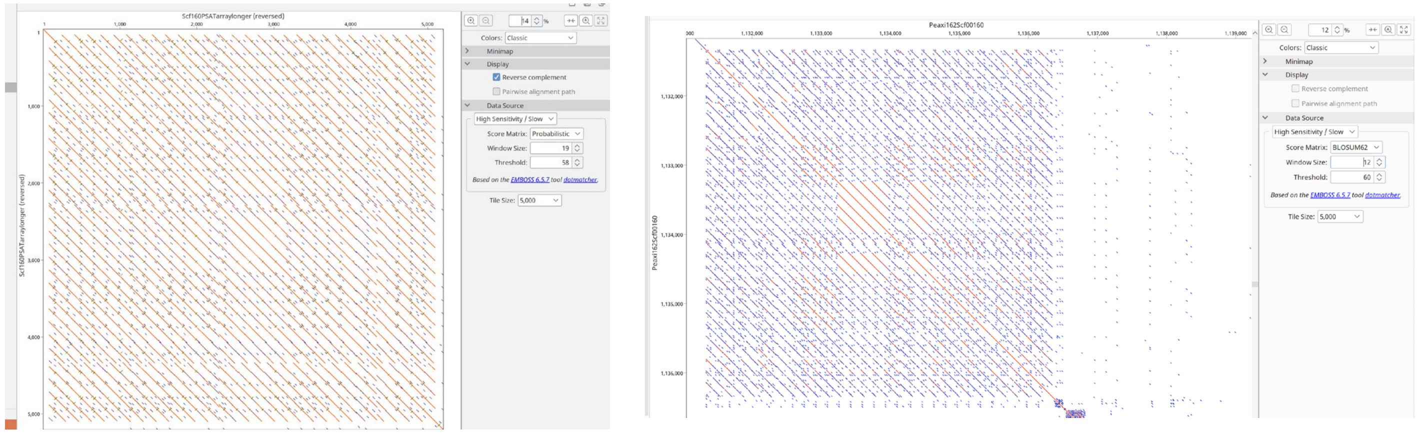

(E)

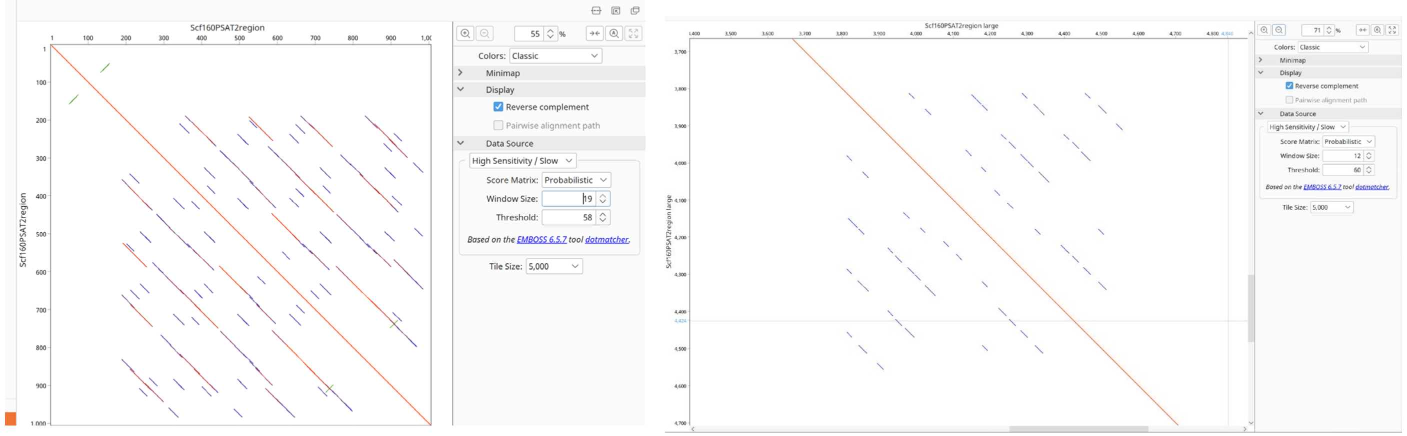

(F)

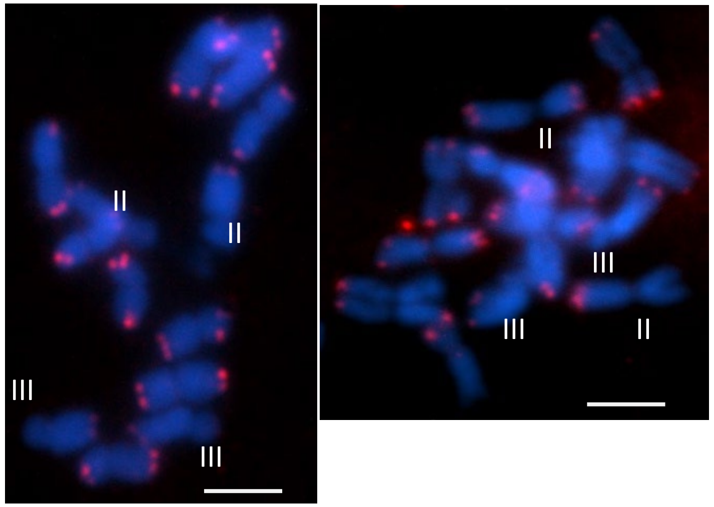

Figure. S3 cont

(D,E) PaxiSAT1 arrays in Peaxi126Scf0160 nt 1,125,000 to 1,140,000 (see Fig. 3). Dotplots are generated with different stringencies as indicated by the setting of the right; (D) Enlargement of the PaxiSAT1 array with 29 monomers of 166-170bp showing a core of highly homologous sequences surrounded by less homologous units; (E) Enlargement of the Variant PaxiSAT2 region showing 5 units that are highly heterogenous

(F) PSAT1 FISH in *P. hybrida V26* (left) and W138 (right), bar = 10µm
